# In-situ quantification of microscopic contributions of individual cells to macroscopic wood deformation with synchrotron computed tomography

**DOI:** 10.1038/s41598-020-78028-4

**Published:** 2020-12-10

**Authors:** Sergio J. Sanabria, Franziska Baensch, Michaela Zauner, Peter Niemz

**Affiliations:** 1 Zurich Ultrasound Research and Translation (ZURT), Institute of Diagnostic and Interventional Radiology, University Hospital Zurich, Raemistrasse 100, 8091 Zurich, Switzerland; 2 Bundesanstalt für Materialforschung und -prüfung (BAM), Unter den Eichen 87, 12205 Berlin, Germany; 3 Institute for Building Materials, ETH Zurich, Stefano-Franscini-Platz 6, 8093 Zurich, Switzerland

**Keywords:** Imaging techniques, Forestry, Forestry, Civil engineering, Mechanical engineering, Composites, Imaging techniques

## Abstract

Wood-based composites hold the promise of sustainable construction. Understanding the influence on wood cellular microstructure in the macroscopic mechanical behavior is key for engineering high-performance composites. In this work, we report a novel Individual Cell Tracking (ICT) approach for in-situ quantification of nanometer-scale deformations of individual wood cells during mechanical loading of macroscopic millimeter-scale wood samples. Softwood samples containing > 10^4^ cells were subjected to controlled radial tensile and longitudinal compressive load in a synchrotron radiation micro-computed tomography (SRµCT) setup. Tracheid and wood ray cells were automatically segmented, and their geometric variations were tracked during load. Finally, interactions between microstructure deformations (lumen geometry, cell wall thickness), cellular arrangement (annual growth rings, anisotropy, wood ray presence) with the macroscopic deformation response were investigated. The results provide cellular insight into macroscopic relations, such as anisotropic Poisson effects, and allow direct observation of previously suspected wood ray reinforcing effects. The method is also appropriate for investigation of non-linear deformation effects, such as buckling and deformation recovery after failure, and gives insight into less studied aspects, such as changes in lumen diameter and cell wall thickness during uniaxial load. ICT provides an experimental tool for direct validation of hierarchical mechanical models on real biological composites.

## Introduction

The end of a period of easy resource extraction and the serious ecological problems are challenges for the twenty-first century. Wood is an environmental-friendly material, engineered by nature. Worldwide there exists 4 billion ha forest, representing 531 × 10^9^ m^3^ of growing biomass^1^. The usage of wood as a construction material coupled with integration of wood residues into energy systems can reduce both greenhouse emissions into atmosphere and fossil fuel use, while continuously increasing wood carbon storage^2,3^. High strength-to-weight ratio, durability, easy machining, predictable fire performance and aesthetic appearance provide wood with a competitive edge with respect to classical construction materials such as steel or reinforced concrete^4,5^. Developments in manufacture and a decline volume of large, old-growth timber have seen a technological transition from solid wood elements into highly engineered wood composites, which allow design of application-tailored load-bearing structures^6–8^. A motivating example is the emerging use of wood-based composites as structural members in multi-story residential buildings^9,10^.

**Figure 1:**
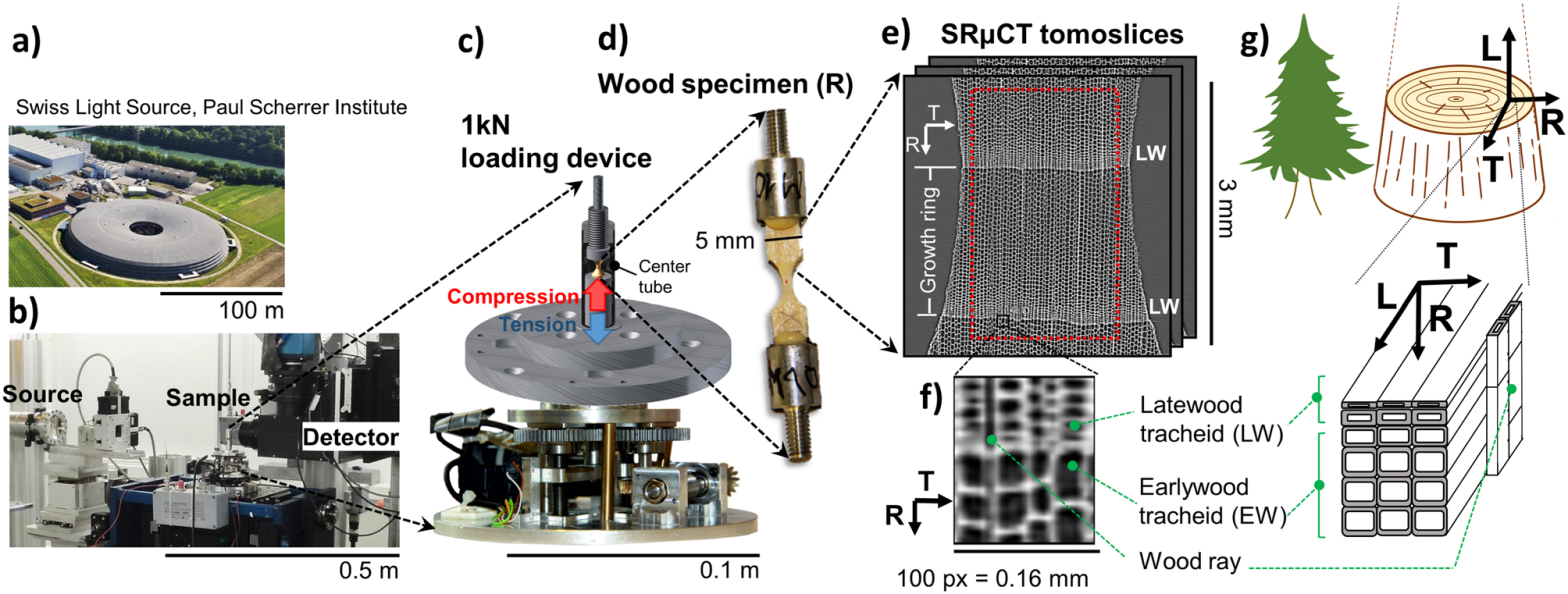
(**a**–**c**) Setup for Synchrotron Radiation Micro-Computed Tomography (SRμCT) of wood. Experiments were performed (**a**) at Swiss Light Source (SLS) of Paul Scherrer Institute, (**b**) at the Tomograhic Microscopy and Coherent Radiology Experiments (TOMCAT) beamline, (**c**) with an integrated uniaxial loading device. For softwood R-specimen (**d**), typical (**e**) tomoslices are shown, which illustrate the periodic (**f**) latewood (LW) and softwood (EW) layers in radial direction (R). The anatomic model (**g**) shows the tubular symmetry of tracheids along the grain direction (L) and of wood rays along R.

Safe exploitation of wood biomass as structural material depends on the correct understanding of its complex mechanical deformation and failure mechanisms. At macroscopic scale, wood is recognized as an orthotropic solid with respect to the three material axes, respectively corresponding to the stem growth direction (grain L), and the radial R and tangential T directions of the seasonal growth rings. At the microscopic scale, wood is a cellular solid, which simplest design is found in softwoods (Fig. 1). They consist of > 90% tube-shaped tracheids, which transport water (cell lumens) and provide mechanical support (cell wall) in the grain direction. Tracheids differ in size and geometry between the thin-walled earlywood cells (EW) formed at the beginning and the thick-walled latewood (LW) cells formed in the latter seasonal growth period. The remaining cells are brick-shaped parenchyma cells that form wood rays, which provide additional reinforcement, as well as nutrient transport and storage in the radial direction; together with < 1% resin canals, which store nutrients and do not influence the mechanical behavior^7,11,12^. Multi-scale mechanical modelling of wood is hindered by this inhomogeneous cellular arrangement, which leads to strong variations of mechanical properties even within samples of the same species^13^.

An improved knowledge of structure-functional relations down to the cellular level is necessary to understand and optimize wood-based components^14^. Mechanical properties of wood derive from the geometry of its cells, and yet current practice is to predict them based on variables, such as bulk density, which do not directly describe the cell structure^15^. Understanding the interactions between micro- and macro-scale allows modeling of mechanical properties^16^, and informs strategies to improve them, for instance, through chemical modification^17^. Best wood species can then be chosen according to mechanical stability for a certain application. Wood-based composite production processes can be optimized^18,19^. New, more damage resistant wood-based products with better performance and improved durability can be designed^20^. Furthermore, the knowledge can be transferred to create new types of materials, for instance, fiber-reinforced polymer-matrix composites in a biomimetic approach^21^, adaptive bioclimatic architecture^22^, or additive manufactured materials^23^. Finally, in the field of production or in-service of wood products, non-destructive testing methods are increasingly important, and basic research is needed to single out suitable monitoring procedures and pave the way for application^24^.

In this context, in situ observation and quantification of wood cellular elements undergoing mechanical deformation is a key experimental goal. Microscopic failure mechanisms in wood have been traditionally visualized with optical and environmental scanning electron microscopy (ESEM)^25^. However, these procedures provide two-dimensional observations of microtome cut slices and show limitations for the monitoring of the small and three-dimensional elastic deformations. Computerized tomography (CT) allows for non-destructive three-dimensional reconstruction of microscopic structures from multiple radiographic observations. In particular, synchrotron radiation micro-computed tomography (SRμCT), which consists of sharply focused and often monochromatic X-rays, achieves sub-micrometer spatial resolution in millimeter-size volumes^26^. The application of SRμCT in wood science is relatively recent, and has progressed from qualitative observation of cellular structures to the computer-assisted quantification of cell geometry, porosity and connectivity^27–32^. SRμCT-compatible mechanical testing devices are also now available^33^, which allow for in situ imaging of the same wood specimen at different deformation states^34,35^ and the visualization of cellular failure mechanisms^36,37^.

The quantification of deformation is one of the most actively studied areas of computer vision, both in biomedical and material science applications^33,38–40^. Image registration finds a geometrical transformation which spatially aligns two images according to a distance measure. Two-dimensional stacks of wood cellular matrices at different moisture-induced swelling states have been aligned with these methods and affine and non-affine deformation components have been quantified^41,42^. Isolated wood cells have also been registered^43^. Digital Volume Correlation (DVC), a three-dimensional extension of Digital Image Correlation (DIC), allows a generalized analysis of three-dimensional deformations in textured CT datasheets. By correlating image subsets within an adjoint search window, local displacement vectors and strain fields are estimated. Commercial tools are available, and the method is popular for the analysis of geomaterials, granular materials or foams, as well as bone and biological soft tissues. It has also been applied to wood cellular structures^34,44^ and to wood-based composites^45,46^. However, it suffers from some limitations. The spatial resolution of the strain estimates is directly related to the correlation subset size^47^, which in the case of wood (100–200 μm) requires tenths of unit cells for a robust tracking^34^. Moreover, the method assumes continuous deformation fields, and can fail at strong strain gradients as typically found upon strong plastic deformation and failure^46^. 3D deformation tracking/registration methods are also computationally expensive, a recent benchmark requiring one day of computation for samples of < 500^3^ voxels^42^.

Particle tracking (PT), on the other hand, is based on the automatic segmentation of discrete elements (e.g. pores, particles) and the tracking of their geometrical changes upon successive deformation states. It currently finds application in the analysis of crack tip morphology during fracture, the analysis of deformation in granular geomaterials or in volumetric flow velocimetry^48–50^. Since PT does not treat the material as a continuum, it can identify, for instance, individual particle rotations and their contribution to the total deformation field^51^. On the other hand, it requires a dedicated image analysis for each application in hand. To the best of our knowledge, PT has not been applied to densely packed cellular matrices, such as wood.

Building from PT concepts, in this work we introduce Individual Cell Tracking (ICT) as a novel approach for automatic deformation measurements at wood microscale.

Miniaturized wood specimens were manufactured from Norway spruce (*Picea abies* [L.] Karst), which cellular microstructure is representative of commonly used wood construction biomass. SRμCT imaging (Fig. 1a,b) of complete wood cellular structure was performed under controlled specimen deformation conditions. For this purpose, a custom-designed uniaxial loading device was installed into the TOMCAT beamline of the Swiss Light Source (SLS) synchrotron light source (Fig. 1c). The wood specimens (Fig. 1d) were mechanically loaded while acquiring in situ SRμCT tomograms at successive deformation states until failure (Fig. 1e).

**Figure 2:**
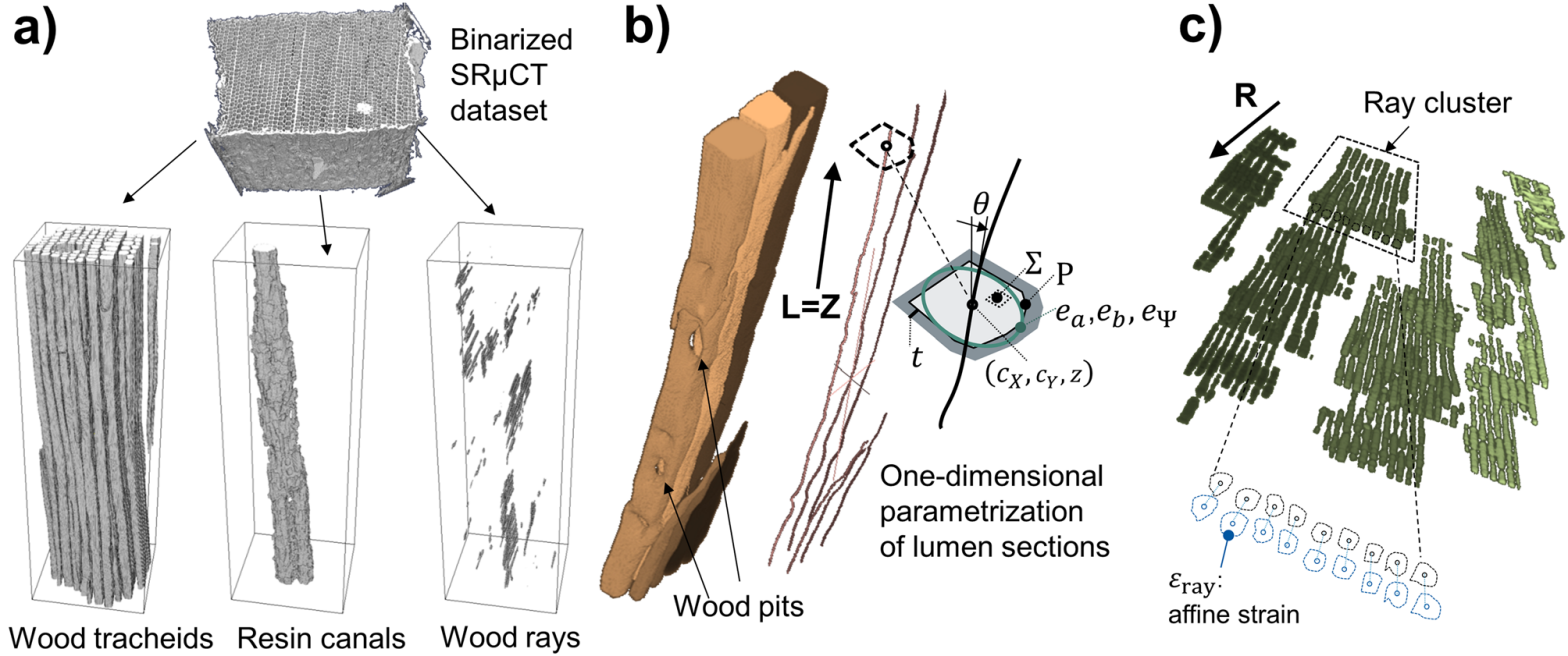
(**a**) Segmentation of wood tracheids, wood cells, and resin canals from SRμCT. The segmentation is performed based on the binarization and morphological analysis of the cell lumens -air voids- which provide approximately closed tubular geometries. (**b**) High-resolution renders of wood tracheids. The split cell structure visualizes in different colors the cell lumens of each tracheid. The tubular cell structure allows one-dimensional parametrization of tracheid lumen cross-sections along the grain direction L. The cell wall thickness *t* is determined from the skeletonization of cell wall substance with respect to the adjacent tracheid cell lumens. Wood pits are preserved during the segmentation process as landmarks for deformation tracking. (**c**) One-dimensional parametrization of wood rays along the radial direction R, including clustering of lumens belonging to the same wood ray. The ray lumen parameterization procedure along R is analogue to the parameterization of tracheid cells along L.

For ICT analysis, first, individual cells of different categories (tracheids, wood rays) (Fig. 1g) were segmented from SRμCT tomograms with 3D morphological operations (Fig. 2). Then, corresponding cell pairs were registered in successive deformation states, the geometrical changes of individual cells were analyzed, and deformation vectors were calculated. The proposed procedure runs automatically over thousands of cells in realistic wood cellular structure and:

- Provides deformation fields at single cell scale spatial resolution,
- Can be applied at high deformation levels, including cell failure, and
- Allows the direct quantification of deformation mechanisms of different cell types (tracheids, wood rays) and their structure–functional relation to the total sample deformation.

**Figure 3:**
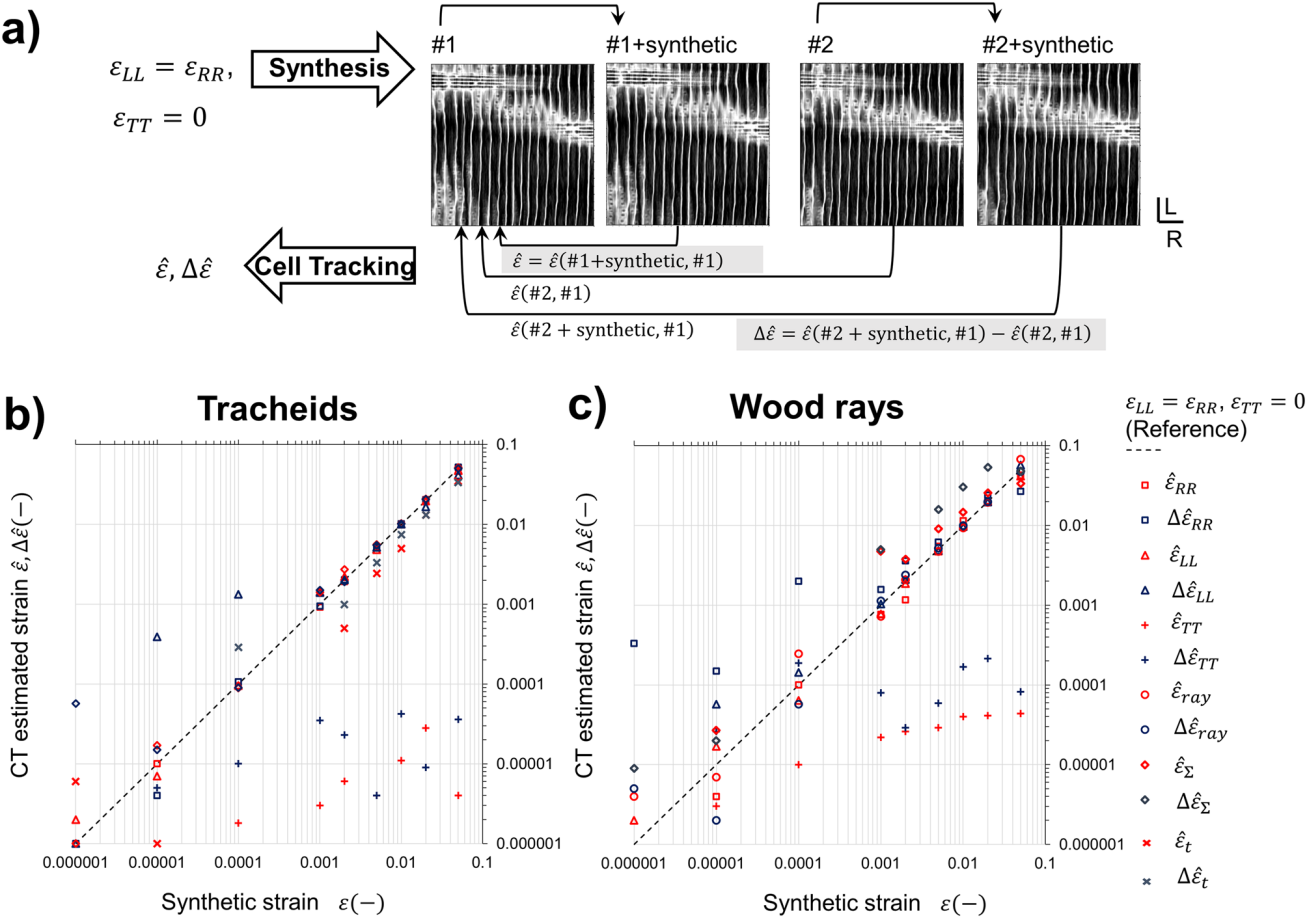
Validation of Individual Cell Tracking (ICT) method by estimation of synthetic deformation fields, which are introduced in experimental softwood SRμCT. (**a**) Data processing workflow for estimation of absolute $\hat{\varepsilon}$ and differential ${\Delta }\hat{\varepsilon}$ accuracy of ICT strain measurements. For absolute accuracy, the same experimental dataset #1 is used as reference and to introduce synthetic deformation. For differential accuracy, deformation is introduced in a second experimental dataset #2, and estimated with respect to #1. Strain estimation results are shown for tracheids (**b**) and wood rays (**c**) for macroscopic strains $\varepsilon_{ii}$, cell lumen $\varepsilon_{{\Sigma }}$ and cell wall $\varepsilon_{t}$ swelling, and wood ray affine strain $\varepsilon_{ray}$. ICT strain estimation error is anisotropic and lowest over the cell cross-section. Consequently, for tracheids, the lowest error is observed in the RT plane, and for wood rays in the LT plane.

**Figure 4:**
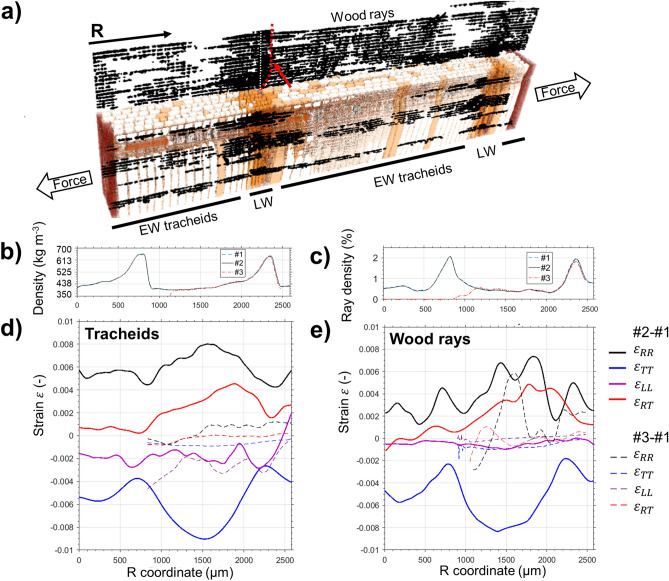
ICT for radial tension experiment (R-specimen). (**a**) Segmentation of tracheids and wood rays in reference state #1. (**b**) Tracheid density profile along a full growth ring. (**c**) Wood ray density profile. (**d**) Average strains calculated from tracheids in function of radial coordinate R. (**e**) Strains calculated from wood rays. For both (**c**) and (**d**), the elastic state #2 shows a heterogenous strain distribution along the growth ring, which is released upon fracture #3. The fracture line occurs at R = 1200 μm, after the first LW/EW transition, and is marked with a dashed red line in (**a**). Wood rays and tracheids follow similar strain patterns, indicating composite deformation.

**Figure 5:**
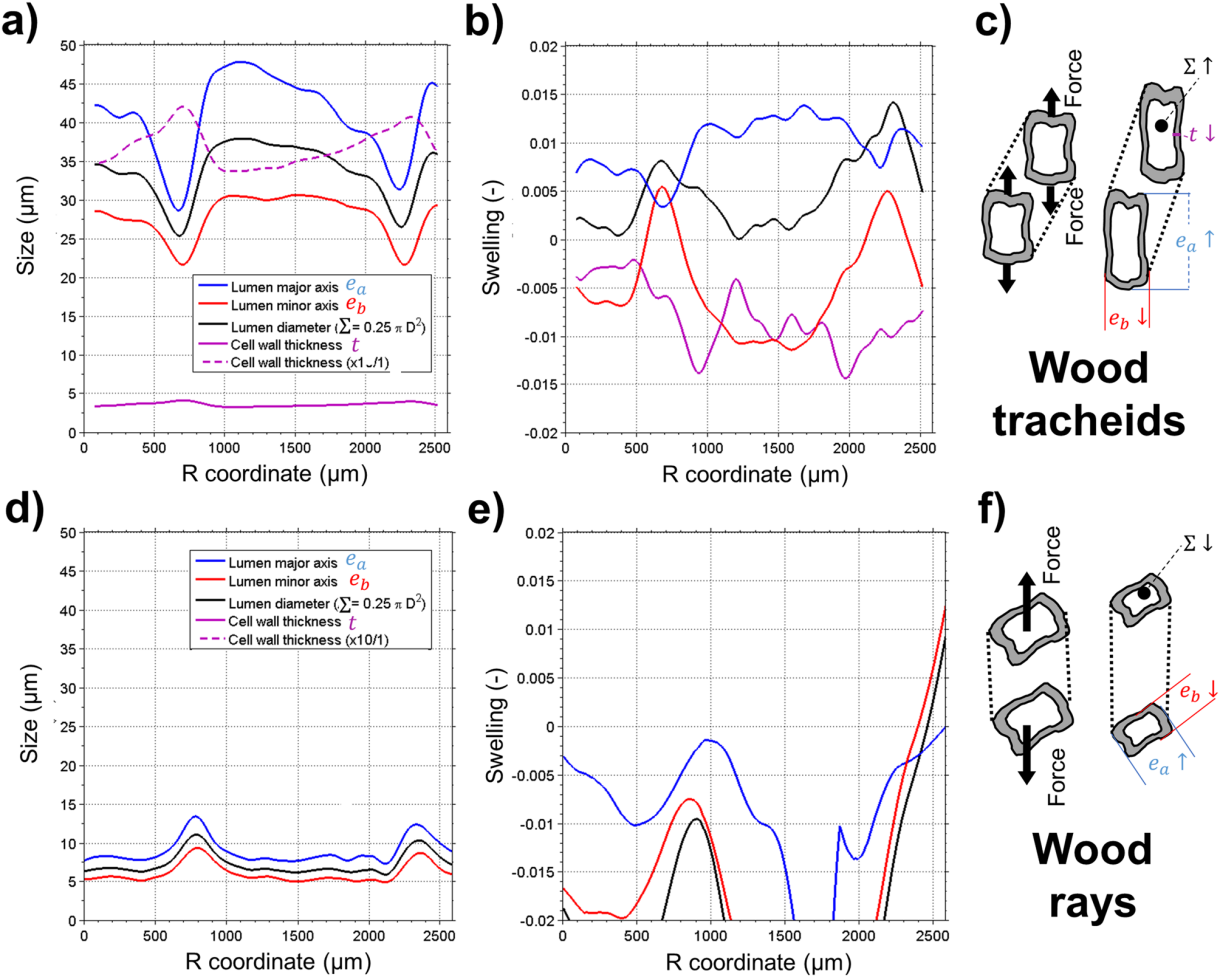
Geometric cell deformation during radial tension experiment (elastic state #2 of Fig. 4). (**a**) shows cell dimensions, (**b**) strains and (**c**) deformation schematic for wood tracheids. Due to tension in R and Poisson effect in T, tracheid lumen’s major axis $e_{a}$ expands and minor axis $e_{b}$ compresses; with strains along the growth ring following $\varepsilon_{RR}$ and $\varepsilon_{TT}$, respectively (Fig. 4). An overall increase of lumen area ${\Sigma }$ is observed, with a corresponding thinning of the cell wall *t*. (**d**) shows cell dimensions, (**e**) strains and (**f**) deformation schematic for wood rays. Due to tension in R and Poisson effect in T and L, both major $e_{a}$ and minor $e_{b}$ cell axes compress, with an overall decrease of lumen area ${\Sigma }$.

**Figure 6:**
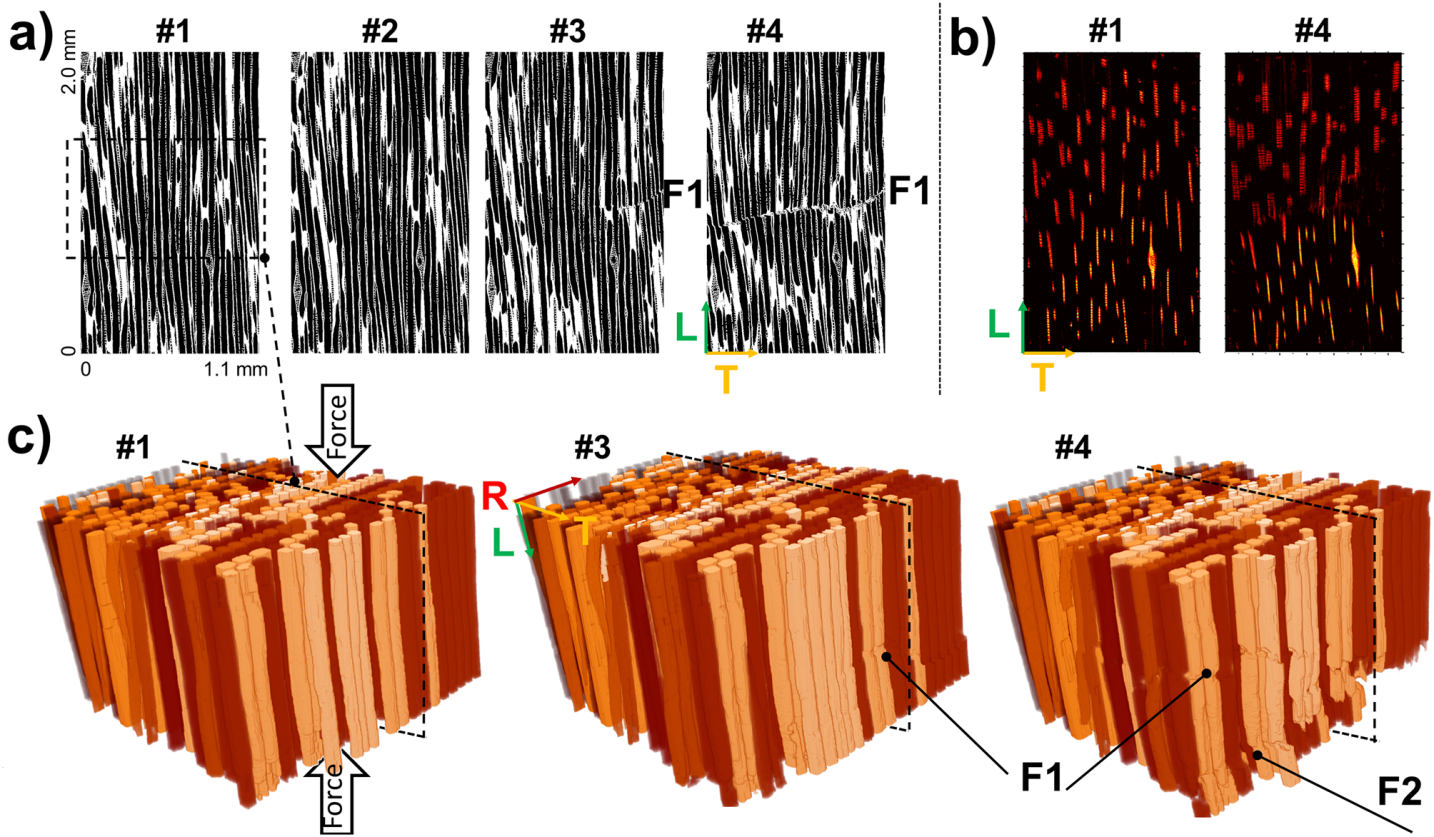
ICT for longitudinal compression experiment (L-specimen). (**a**) In-situ LT slices for deformation states #1 (baseline), #2 (elastic deformation), #3 (plastic onset), and #4 (densification). (**b**) LT projection of segmented wood rays. (**c**) Three-dimensional render of segmented tracheids. Co-registered cells are painted with the same color. In #3, the fracture plane F1 starts developing. With additional compression #4, F1 extends over the specimen cross-section and to an additional plane F2.

**Figure 7:**
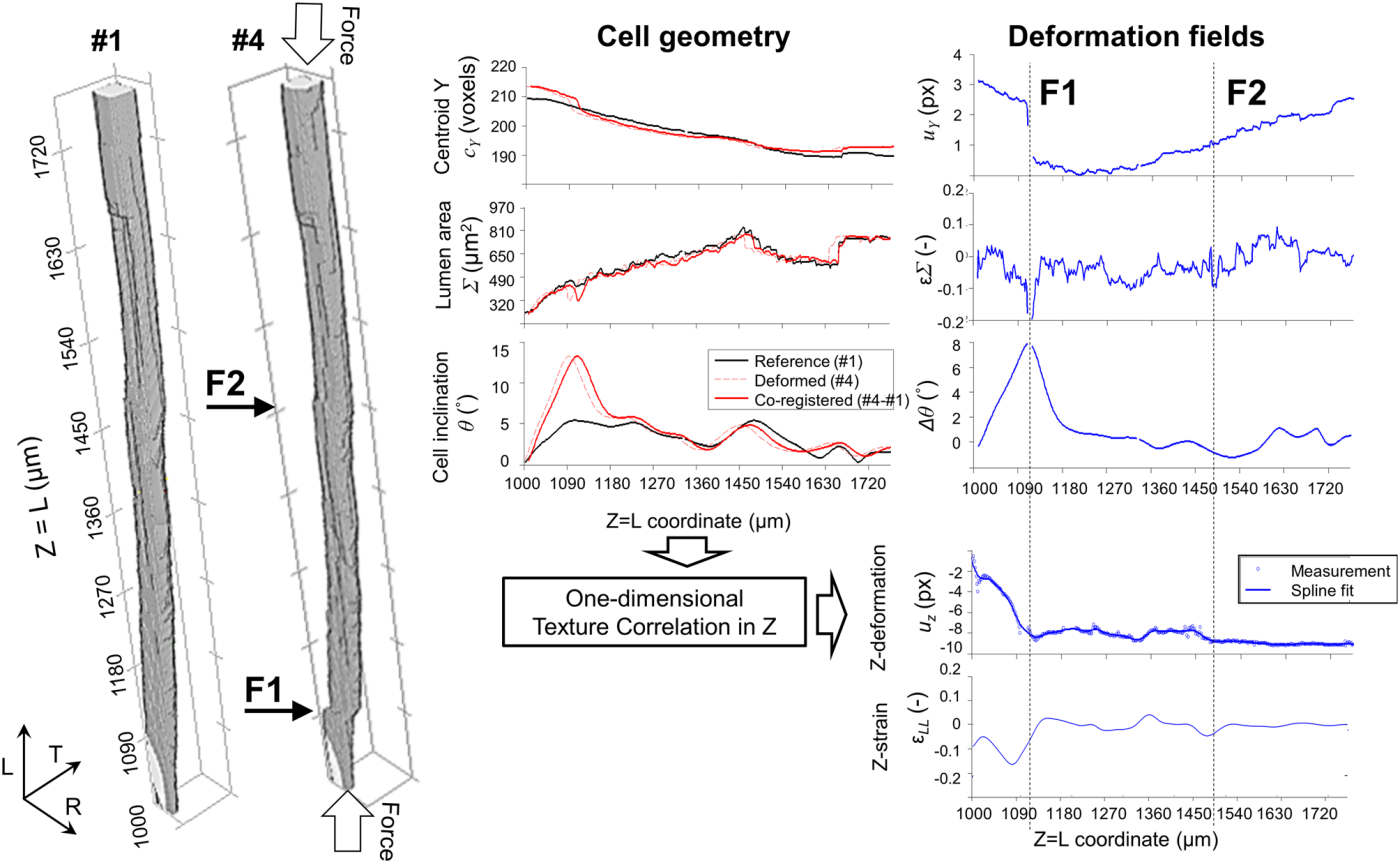
Individual Cell Tracking (ICT) method for single tracheid of L-specimen subject to compression. Based on one-dimensional cell parametrization (Fig. 2), cell geometry parameters are obtained for reference (#1) and densification (#4) states and co-registered with texture correlation in Z. The deformation fields reveal two fracture plane F1 and F2. Buckling (${\Delta }\theta > 0$) is observed in F1, while for this cell F2 shows telescopic shortening ($\varepsilon_{LL} < 0,\varepsilon_{{\Sigma }} < 0$) only.

**Figure 8:**
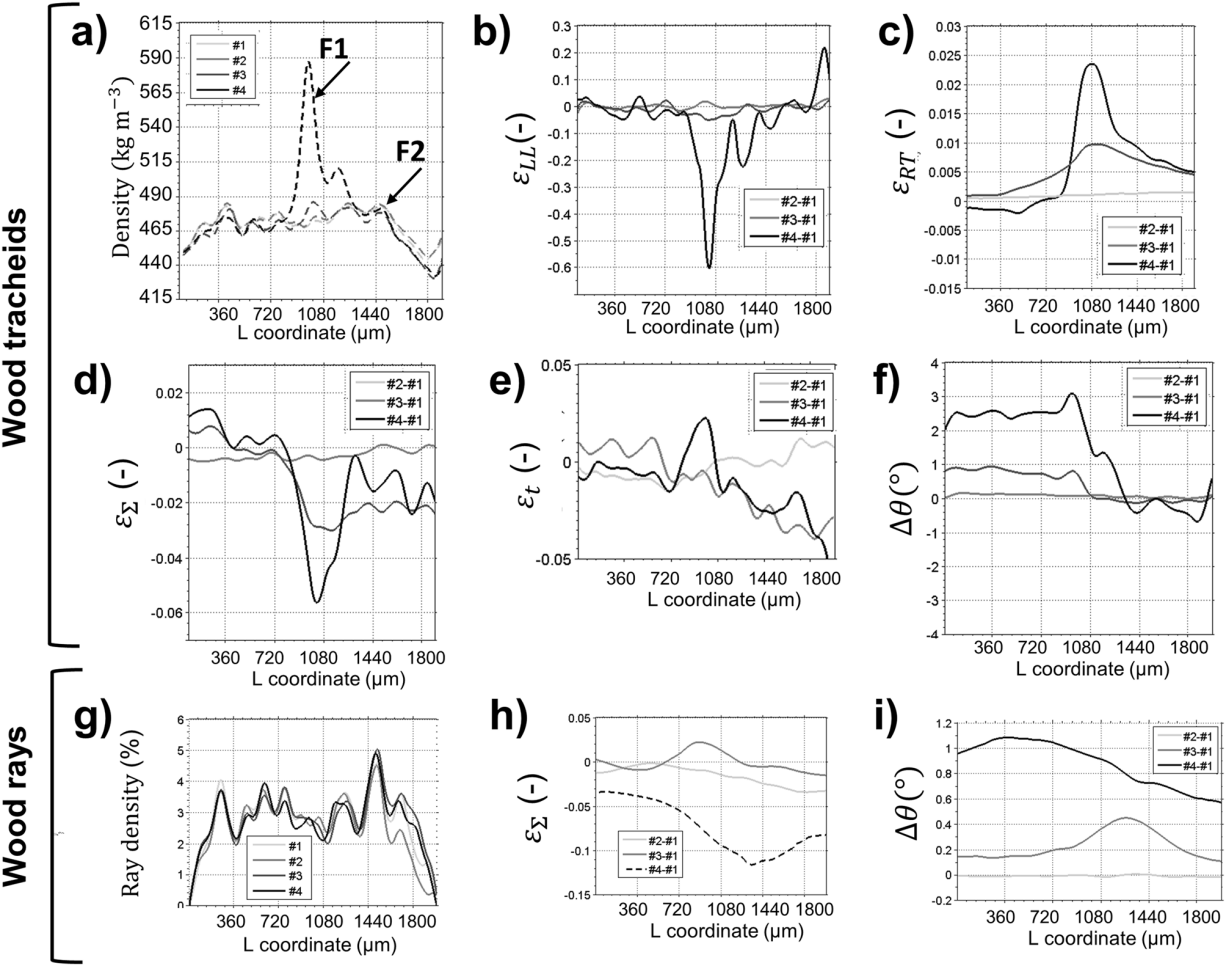
Average deformation profiles along grain direction for L-sample of Fig. 6. (**a**–**f**) are computed with ICT of tracheids, (**g**–**i**) with ICT of wood rays. Macroscopic strains $\varepsilon_{LL} ,\varepsilon_{RT}$ and geometric cell deformations (cell lumen area $\varepsilon_{{\Sigma }}$, cell wall thickness $\varepsilon_{{\text{t}}}$, cell inclination ${\Delta }\theta$) are shown. Strong densification effects are observed for wood tracheids concentrated in fracture lines F1 and F2, while wood rays show more distributed deformation patterns. $\varepsilon_{{\Sigma }} , \varepsilon_{RT}$ and ${\Delta }\theta$ provide clear indications for the onset of plastic deformation (#3), while macroscopic density and compression $\varepsilon_{LL}$ are less apparent.

In Sect. Deformation measurement accuracy, we quantify the strain resolution of the new method and demonstrate its applicability for the three-dimensional deformation analysis (Fig. 3). Next, in Sects. Multi-scale deformation analysis of growth ring loaded radially in tension until fracture and Multi-scale analysis of softwood compressed longitudinally until densification we describe in situ investigations of anisotropic tension and compression from small elastic deformation until failure. Two representative experiments were carried out: tension load in radial direction until fracture (R-specimen) (Figs. 4, 5), and compression in grain direction until wood densification (L-specimen) (Figs. 6, 7, 8). By visualizing spatial deformations distributions calculated independently from individual cell categories and by performing correlation analysis, we correlate macroscopic and microscopic deformation features and investigate structure–function interactions between different cell types.

## Results and discussion

### Deformation measurement accuracy

To evaluate the accuracy of ICT, synthetic deformation fields were added to the R-specimen datasets (Fig. 3a). For this purpose, constant strain was simultaneously introduced in both R and L directions ($\varepsilon_{RR}$,${ }\varepsilon_{LL}$). *Absolute accuracy* $\hat{\varepsilon }$ was measured by adding synthetic deformation to the reference state #1 (#1 + synthetic), and then measuring with ICT strain of #1 + synthetic with respect to #1. *Differential accuracy* ${\Delta }\hat{\varepsilon }$ was measured by adding synthetic deformation to the deformed state #2 (#2 + synthetic), measuring with ICT strain of #2 + synthetic with respect to the reference state #1, and finally subtracting the ICT measured strain between #2 and #1.

Figure 3 shows ICT strain estimates for tracheids and wood rays. The accuracy is highest along the cell-cross section (RT for tracheids, LT for wood rays) and lowest in the cell longitudinal direction (L for tracheids, R for wood rays). Due to the tubular cell geometry (Fig. 2b,c) of both cell types, tracking is more challenging in the longitudinal cell axis, for which symmetry reduces the available landmarks (for instance, wood pits in Fig. 2) and deformation tracking accuracy. *Absolute accuracy* is limited by both cell segmentation and deformation parametrization. *Differential* accuracy is further influenced by experimental uncertainties between #1 and #2 acquisitions, such as vibration artifacts and sample relaxation strains. While absolute and differential accuracy are similar in cell-cross sections, differential accuracy is reduced with respect to absolute accuracy along the longitudinal cell axis. The sensitivity limit for strain measurements in tracheids is $\varepsilon_{RR}$ < 5 × 10^–5^ and $\varepsilon_{LL}$ < 2 × 10^–3^, whereas for wood rays $\varepsilon_{LL}$ < 2 × 10^–4^ and $\varepsilon_{RR}$ < 2 × 10^–3^. By evaluating deformations at individual cell level, ICT optimizes strain tracking accuracy, since $\varepsilon_{LL}$ estimates from wood rays are not influenced by the lower $\varepsilon_{LL}$ accuracy of adjacent wood tracheids and vice versa for $\varepsilon_{RR}$. Since the average tracheid lumen diameter is 35 µm, $\varepsilon_{RR}$ < 5 × 10^–5^ corresponds to a deformation of 1.8 nm, i.e. three orders of magnitude below the voxel size. A similar precision is observed for tracking changes in the tracheid lumen area $\varepsilon_{\Sigma }$ < 5 × 10^–5^. Tracheid cell wall thickness deformation down to $\varepsilon_{t}$ < 5 × 10^–3^ also follows accurately the synthetic strain trends. This high sensitivity to small strains is explained by the fact that ICT computes strains using global metrics (lumen centroid, area and inclination), which average the contributions of all lumen voxels, thereby reducing the effect of discretization noise. Due to the smaller lumen diameters, wood ray lumen area deformation can presently only be tracked down to $\varepsilon_{\Sigma }$ < 5 × 10^–3^. Similarly, for wood rays, due to the small lumen size and the simultaneous presence of multiple cell lumens per cluster, cell wall deformation analysis is more involved, and was left out of the scope of this work.

The tracking accuracy obtained with ICT is comparable or superior to values reported for DVC, for which typical displacement uncertainties are 0.1–0.01 voxels ^33^. Gillard et al.^52^ measured strains in bone with DVC down to 2 × 10^–4^–8 × 10^–4^. As for lateral resolution, minimum DVC correlation subsets to track displacements in wood have been reported in the range 110–330 µm, i.e. 3–9 tracheid diameters or 16–47 wood ray diameters^34^. ICT, on the other hand, operates at single cellular level.

### Multi-scale deformation analysis of growth ring loaded radially in tension until fracture

#### Macroscopic deformation with independent analysis of tracheids and wood rays

Figure 4a shows a 3D segmentation of the R-specimen, corresponding to the region of interest of (R, T, L) = (2.6, 1.5, 1.8) mm marked in Fig. 1e. Out of the segmented 1.67 × 10^9^ voxels, 3700 tracheids were automatically detected in first iteration (67.9% of void voxels), with 232 additional ones (all LW cells) in second iteration. 312 wood rays were detected, which account for ~ 3% void voxels. Figure 4b shows a density profile along R, which was calculated based on the cell wall amount of tracheid cells. Discrepancies with the specimen’s gravimetric density (400 kg m^−3^ ± 60 kg m^−3^) are explained by the natural density variability of wood and the small sample dimensions.

A growth ring (thickness 1500 µm) is defined by two sharp LW–EW discontinuities at R = 850 µm and R = 2350 µm, corresponding to the start and end of the growth season. The density increases gradually between EW and LW cells. Deformation state #2 (elastic) shows a barely visible 20 µm displacement of the density profile with respect to #1 (preload). The fracture line (density drop in state #3) occurs at 20% of the growth ring width within the EW region (R = 1150 µm). For comparison, the fracture line is also marked with an dashed red line and an arrow in Fig. 4a. Figure 4c shows the wood ray density (percentage of wood ray voxels) across the growth ring (0.5–2%). As it is also visible in Fig. 4a, wood rays extend in R direction over several growth rings. Wood ray density increases smoothly from EW and LW cells. Opposite to tracheids, it does not show a sharp LW–EW discontinuity, but reduces gradually until R = 1200 µm, where the fracture line (#3) occurs. Deformation #2 is visible as a displacement of the density profile.

Figure 4d shows macroscopic strains calculated with ICT by analyzing tracheids. The elastic deformation #2 induces heterogeneous strains along the growth ring. Radial strain $\varepsilon_{RR}$ shows a peak of 0.8% at mid-width of the growth ring (R = 1600 µm) with respect to a minimum $\varepsilon_{RR}$ of 0.4% at the LW region. $\varepsilon_{RR}$ is accompanied by an approximately proportional compressive tangential strain $\varepsilon_{TT}$, with average Poisson ratio $v_{TR} = - \frac{{\varepsilon_{TT} }}{{\varepsilon_{RR} }} = 0.94$. ${ }\varepsilon_{TT}$ shows a smoother pattern than $\varepsilon_{RR}$, and $v_{TR}$ varies between 0.69 (LW) to 1.25 (R = 1300 µm). Significant shear strains $\varepsilon_{RT}$ up to 0.5% are observed in the RT plane, with a similar trend to $\varepsilon_{RR}$. Figure 4e shows strains calculated with ICT by analyzing wood rays. The strains are consistent with Fig. 4d, suggesting that wood tracheids and wood rays deform together as a single composite unit. $\varepsilon_{RR}$ and $\varepsilon_{TT}$ are slightly lower (0.2%) than for tracheids, which indicates a deformation restraining effect in R direction by wood rays. $\varepsilon_{LL}$ is well-resolved based on wood rays, with $v_{LR} = - \frac{{\varepsilon_{LL} }}{{\varepsilon_{RR} }} = 0.07$. Fracture state #3 leads to deformation release for both tracheids and wood rays, with most of strain of state #2 vanishing. Strains $\varepsilon_{RR}$ and $\varepsilon_{TT}$ are reduced below 0.1%, and $\varepsilon_{LL}$ below 0.02%. Both $\varepsilon_{LL}$ calculated based on tracheids and $\varepsilon_{RR}$ calculated based on wood rays show noisy patterns, which are explained by the lower accuracy of the ICT method along the longitudinal cell axes (see Sect. Deformation measurement accuracy, Fig. 3).

Farruggia et al.^53^ found $v_{TR} > 1$ in microtensile tests of EW, with smaller values in LW, in agreement with our values. The heterogeneous $\varepsilon_{RR} ,{ }\varepsilon_{RT}$ pattern across the growth ring observed in this work resemble moisture-induced free swelling in softwood, with anisotropy ratio $\varepsilon_{RR} {/}\varepsilon_{TT}$ increasing at mid-growth ring^54,55^. Yet, for moisture-induced strain, $\varepsilon_{TT}$ shows a constant pattern and $\varepsilon_{RR}$ peaks closer to the LW region. On a constrained unit cell, Rafsanjani et al.^56^ found highest hygroscopic R displacements at mid-width of the growth ring, with the stiffer LW pushing radially the softer EW. Jernkvist et al.^57^ optically measured deformation in softwood growth rings loaded in tension and found $\varepsilon_{RR}$ and $\varepsilon_{TT}$ peaking at mid-growth ring width and with a 50% decrease at LW–EW transition, together with significant $\varepsilon_{RT}$, in close agreement with our observations. These were explained by the apparent stiffening of the softer EW by the sharp interface to the significantly stiffer LW region. Similar trends were observed by Moden et al.^58^, who found less pronounced strain variations than in the growth ring density profiles. This is associated to the constraining between adjacent cells. $\varepsilon_{RR}$ is more heterogeneous than $\varepsilon_{TT}$. A possible explanation is that EW and LW cells act as a parallel system of strings in T direction, deforming as a single composite unit, while in R direction they act as a series system of elastic strings, thus allowing for heterogeneous strain behavior across cells.

We observed deformation release after fracture and no significant strain close to fracture surface, indicating fast failure and small plastic deformation. Fracture occurs in the EW region, which is structurally weaker than LW^59^. Wood rays act as a reinforcement preventing crack propagation^60^. A previous fractographic analysis of radially tensioned wood samples^36^ associated radial failure to the weaker ray section. Accordingly, we observed a smooth decrease of wood ray lumen diameter within the EW region, which roughly corresponded to the position of the fracture plane.

#### Microstructure deformations at subcellular scale

Figure 5a,b quantify the geometric deformation of wood tracheids in function of the growth ring position. The tracheid lumen size ranges between 22 and 48 µm, with smallest values at LW. The lumen geometry is anisotropic in EW (aspect ratio R/T = 1.6) and more isotropic in LW (aspect ratio R/T = 1.3). The cell wall thickness ranges between 3.4 and 4.2 µm, with thinnest values in EW (Fig. 5a). The lumen deformation (Fig. 5b) follows the macroscopic strain patterns of Fig. 4d, with the major axis following $$\varepsilon_{RR}$$ and the minor axis following $$\varepsilon_{TT}$$. In the LW region, $$v_{TR} < 1 $$ and the cell lumen accordingly shows a surface increase $$\varepsilon_{\sum } =$$ 1.1%. In EW at peak $$\varepsilon_{RR}$$ strain, the cell lumen surface does not change ($$\varepsilon_{\sum } \approx$$ 0%) confirming the macroscopic observation $$v_{TR} \approx 1$$. The tension load stretches the cell wall, reducing its thickness in a range $$\varepsilon_{CW} =$$[− 0.3%, − 1.4%]. Even at EW positions where $$\varepsilon_{\sum } \approx$$ 0%, due to the cell stretch along R the lumen perimeter increases and consequently the cell wall thickness is reduced. Figure 5d,e quantify the deformation of wood rays. The lumen size ranges between 5 and 13 µm (Fig. 5d). Opposite to wood tracheids, for wood rays the largest lumen sizes are observed in LW. The radial load induces an overall compression of the lumen size ($$\varepsilon_{\sum }$$ = 2%) for both major and minor cell axes (Fig. 5d). The compression is largest in the EW region, where the R strain of the wood rays is highest, thereby showing a Poisson effect in both T and L directions.

The ICT method allows direct visualization of the subtle geometric contributions of different cell types to the global deformation state. Upon tensile deformation, we quantified a thinning of the cell wall thickness of around 0.8% accompanied by a similar increase of the lumen section, which accounts for a detection of 28 nm deformation in a 3.5 µm thick cell wall. Cell wall stretching upon tensile deformation has been previously hypothesized based on the observed linear dependence of elastic modulus with density over a broad range of wood species^58^. The lumen area remained unchanged at the center of the growth ring, with approximately equal opposed deformation of the major and minor lumen axes, allowing geometric observation of Poisson ratio $v_{TR} \approx 1$.

#### Correlations between microscopic and macroscopic deformation properties

Voxel-wise correlation coefficients *r* between deformation parameters for elastic deformation state #2 are provided in Appendix B as Supplementary Tables S1–S3. All *r* > 0.001 are significant (*p* < 0.05). Due to Poisson effect, $\varepsilon_{RR}$ shows a negative correlation with $\varepsilon_{TT}$ (*r* = − 0.639), and $\varepsilon_{TT}$ positively correlates with $\varepsilon_{LL}$ (0.372). From all calculated strain fields, $\varepsilon_{TT}$ calculated with both wood rays and tracheids show the strongest agreement (0.835). Shear strain $\varepsilon_{RT}$ increases at larger deformations $\varepsilon_{RR}$ (0.225).

As for geometric wood tracheid deformation, $\varepsilon_{RT}$ is correlated with shifts in lumen orientation $\Delta e_{{\Psi }}$ (− 0.312). Strain $\varepsilon_{RR}$ increases for larger tracheid cell lumens ${\Sigma }$ (0.166) and thinner cell walls $t$ (− 0.229), both characteristic of EW cells. The correlation with cell size is strongest for $\varepsilon_{TT}$, with *r* = − 0.262 for ${\Sigma }$ and *r* = 0.457 for *t*. The cell wall *t* is also negatively correlated with tracheid inclination $\theta$ (− 0.459), while $\varepsilon_{RR}$ increases with $\theta$ (0.179). The cell wall swelling $\varepsilon_{t}$ is negatively correlated with increase of lumen size $\varepsilon_{{\Sigma }}$ (*r* = − 0.344*)*.

As for geometric wood ray deformation, $\varepsilon_{RT}$ is correlated with shifts in ray cell inclination $\Delta \theta$ (*r* = − 0.512). Tracheid lumen areas ${\Sigma }$ are correlated with wood ray inclination $\theta$. Poisson compression strain $\varepsilon_{LL}$ results in ray cluster compression $\varepsilon_{ray}$ (*r* = 0.259), which reduces the inner distances between ray lumens, and a reduction of ray lumen size $\varepsilon_{{\Sigma }}$ (0.237). Larger ray lumen areas ${\Sigma }$ are correlated with larger adjacent tracheid cell walls *t* (r = 0.383), as found in LW regions, and show smaller deformations, with *r* = − 0.355 for $\varepsilon_{RR}$.

As the distance to wood rays $d_{ray}$ increases, the strains $\varepsilon_{RR}$ (0.052) and $\varepsilon_{TT}$ (− 0.108) significantly increase in absolute terms, and the tracheid inclination $\theta$ is reduced (− 0.060).

Correlation analysis reveals larger deformations for thinner cell walls *t* and larger lumen areas, which are characteristic of EW cells. It is known from previous work that LW and EW differentiate in their chemical and nanoscopic building. For instance, the microfibril angle varies from LW and EW, and the S2-layer reduces in thickness from LW and EW, which leads to different amount of lignin/cellulose over the complete cell wall and stiffer LW than EW cells^61^.

#### The role of wood rays in wood composite structure: comparison with previous studies

Wood rays have been associated to both nutrient transport and storage and to adaptive tree reinforcement in radial direction. Burgert and Eckstein isolated single wood ray cells and subjected them to microtensile tests^62^. They found significantly higher strength in isolated wood ray cells than in the macroscopic wood material, which verified the radial reinforcement of wood rays^63^. Reiterer et al.^60^ and Burgert et al.^64^ analyzed the macroscopic effect of wood rays by identifying improved radial strength properties in wood species with similar wood structure except for the distribution of wood ray cells. Similarly, Elaieb et al.^65^ recently linked wood ray presence with hygroscopic stability, by showing significant correlations across species between microscopic wood ray proportion and reduced drying-induced macroscopic shrinkage. The functional adaption of wood rays has been investigated by subjecting growing trees to radial loads^12^, and analyzing wood microstructure and macroscopic mechanical properties at different stem locations. Wood rays act as stiff radial pins to prevent shear slipping of EW and LW layers during stem bending due to wind loads^12^.

Our work for the first time directly measures the mechanical deformation of wood rays embedded within a tissue composite structure. In real wood composites, due to the cells not being isolated, they influence and are influenced by the surrounding cellular microstructure. In softwoods, wood rays are small and difficult to isolate; in our experiments they accounted for only 3% of total material volume. We observed in 2.2.1 that wood rays followed similar deformation trends to wood tracheids, showing that interactions between both cell types result in macroscopic composite deformation. The strain of wood rays is locally smaller than in tracheids, showing a less compliant behavior. The negative correlation between distance to wood rays and strain shows the reinforcement effect of wood rays. Accordingly, Xue et al.^66^ found in cryomicrotome sections of hardwood largest shrinkages for cells most separated from wood rays.

The presence of wood rays affected the inclination of wood tracheids $\theta$, which wrap around the wood ray cells. Similar weaving effects were observed by^28^ for hardwood vessels cells. Changes in $\theta$ are known to affect mechanical properties in grain direction^67^. The radial stretching of wood rays was observed to reduce their lumen sections, showing a Poisson effect at cellular scale. Ray cells within a single ray cluster also modified their relative L-distances when pulled in radial direction. Together with the heterogeneous EW/LW cell microstructure, the geometric imbalances resulting from the embedding of tracheids and wood rays in the same composite structure, introduce shifts in tracheid lumen tension and wood ray inclination, which result in shear strains during the tension experiment.

The role of wood rays in wood fracture has also been indirectly analyzed with microscopic and micro-tomography methods, which allow visualizing cell distribution in fracture surfaces^36,60^. Baensch et al.^36^ associated radial wood failure with the weakest ray sections. Similarly, we observed smallest ray lumen diameters in earlywood at the position of the fracture plane. Reiterer et al.^60^ found that, for similar number of wood rays in a volume, wood species with larger wood rays show higher radial stiffness properties. Accordingly, we observed that ray lumens were larger surfaces were less compliant, showing smaller radial strains. Overall, our results suggest that wood ray with larger lumens are mechanically stronger.

### Multi-scale analysis of softwood compressed longitudinally until densification

Despite large non-linear deformations due to cell densification, ICT successfully co-registers individual tracheids and wood rays in specimen L between successive compression states (Fig. 6). A main densification line develops at the sample mid-point (F1), with a secondary densification line being visible at the bottom of the sample (F2). Figure 7 exemplifies the data evaluation process of the geometrical changes of a single tracheid from reference #1 to densified state #4. F1 is visible by eye comparing the renders before (state *#1*) and after (state #4) densification. Longitudinal compression strain ($\varepsilon_{zz}$ < 0) and a reduction of the tracheid section area ($\varepsilon_{\sum }$ < 0) at the compression plane are observed. Moreover, a shear shift in the tracheid lumen centroid ($\Delta c_{Y}$) and an increase of the tracheid inclination (∆θ > 0) were assesed below the compression plane, which indicate cell buckling. F2 is not visible by eye in the segmented datasets but can be identified in the deformation fields with simultaneous $\varepsilon_{zz}$ < 0 and $\varepsilon_{\sum }$ < 0 at the compression plane, which indicate cell densification. For further verification of F2, Contrast to Noise Ratio calculations are included in Supplementary Materials—Appendix C (Figure S2), where F1 shows a CNR of 11.5, followed by F2 with a CNR of 4.7. Since no shear effects are present in F2, the deformation is interpreted as telescopic shortening^37^.

Even if the deformation effects are small for single cells, the ICT method automatically analyzes thousands of cells, revealing consistent deformation patterns. Accordingly, Fig. 8 shows profiles of the average deformation fields along the grain direction L. For state #4, an increase of density (Fig. 8a) is observed at L = 940 µm and L = 1510 µm, corresponding to compression planes F1 and F2, with a third intermediate plane F3 (L = 1150 µm) connecting F1 and F2. The compression planes are characterized by peak compression strains $\varepsilon_{LL}$ = − 60% (Fig. 8b). Densification is also characterized by an increase of cross-grain shear strain $\varepsilon_{RT}$ above F1 (Fig. 8c), which indicates cell buckling.

The ICT method allows visualization of the compression process at the microstructural level. A reduction of the tracheid lumen area $\varepsilon_{\sum }$ < 0 (Fig. 8d) and an increase in cell wall thickness $\varepsilon_{t} >$ 0 (Fig. 8e) indicate densification, which was concentrated at F1 and distributed more homogeneously over the specimen top part. The bottom part of the specimen below F1 was slanted at a constant tracheid inclination ∆θ = 2.5° and not compressed (Fig. 8f). The densification was clearly dominant in plane F1, but buckling effects propagated to top sample planes F2 and F3 as the deformation increased.

Wood rays show more distributed deformation patterns than tracheids. A localized increase of ray density at F1 was not observed (Fig. 8g) and the cell lumen compression was more distributed over the top part of the specimen (Fig. 8h). Finally, the wood ray inclination (Fig. 8i) was not significantly affected by the densification process. These overall shows that wood rays are less subjected to densification than wood tracheids, acting as stiff pins within the composite.

The analysis of deformation states #2 and #3 shows which parameters are more sensitive for small linear deformations (#2) and the onset of plastic deformation (#3). At state #2, deformation is only clearly visualized with $\varepsilon_{\sum } = 0.4\%$ and $\varepsilon_{RT} = 0.001$. Small deformations were homogeneously distributed over the sample length, with a gradual increase of cell RT shearing. In deformation state #3, the development of densification line F1 becomes clearly visible through ∆θ = 1°, $\varepsilon_{\sum }$ = 2% and $\varepsilon_{RT} = 0.01$. $\varepsilon_{LL}$ is less sensitive than the aforementioned parameters and shows a noisy pattern which does not reveal clearly the densification process even at state #3, where $\varepsilon_{LL} = 5\%$.

In early compression stages, deformation is homogeneously distributed over the tracheid length, whereas with higher compression they become localized around fracture lines, which absorb the compressive stresses. At the latest densification stage, collapse of tracheid lumens occur, which increase the macroscopic density. Wood rays behaved as stiff pins, showing more distributed compression patterns, and their inclination was less affected by the densification process. At early deformation stages, deformation was only visible by the buildup of shear strain and small variations of the tracheid lumen section. With larger deformation, shear build up progressed towards cell buckling. In accordance with these observations, Zauner et al.^37^ observed the failure area of spruce wood after uniaxial compression and identified changes of fracture line progression when crossing wood rays. In final densification stages, wood ray cells buckle and are compressed.

## Conclusions

### Individual cell tracking method

This work presents an Individual Cell Tracking (ICT) approach to track deformations in single cells of a macroscopic wood sample. The presented method is a valuable tool for understanding the interactions between microscopic cellular and macroscopic wood composite deformation, and for analyzing the structure–functional relations of different cell types, which can complement conventional macroscopic deformation tracking approaches. This work can inspire research for systematically characterizing the micro-structural mechanical behavior of a wider pallet of wood species than is currently used in wood architecture. The progress in functional wood-inspired materials also suggests that smart combinations of wood species and cellular components can be envisioned—modified or not with chemical or genetic processes. Currently, there are several microstructural models available based on density distributions extracted from tomographic measurements^68–71^, or segmentation of different cell types^72^. ICT may contribute to validate deformation fields provided by these models. A key contribution of ICT is that it enables separation of deformations and analysis of the structure–functional relationships for different cell types. ICT may therefore provide an experimental basis for inferring mechanical material properties of constituent cell components based on inverse FE models of macroscopic deformation observations for defined loads. Inverse FE models are for instance nowadays performed regularly in medical elastography^73^.

The computational framework presented here can be extended to other hierarchical structured composites, for instance wood-based materials (plywood, fiberboard, particleboards…) and other cellular materials, for instance, trabecular bone^74^. Understanding interactions between microstructure and macroscopic mechanical behavior is key to engineer high-performance wood composites. High-resolution tomography observations of wood micro-structure based on ICT can also help to understand and optimize the effect of wood modification processes on mechanical performance^75^.

### Experimental observation of interactions between microscopic deformation, cellular arrangement and macroscopic deformation response of wood

The overall picture of wood’s mechanical behavior that emerges from our experiments is of a composite deforming as a single unit, with both tracheids and wood rays contributing to the macroscopic mechanical behavior. Local heterogeneities are caused by the geometric embedding of tracheids and wood rays within the same composite structure.

Multi-scale deformation analysis of an annual growth ring, which was loaded radially in tension, revealed heterogenous strain distributions. Due to the constrained composite structure, the stiffer LW cells push radially the softer EW cells, leading to a strain peak at mid-width of the growth ring. Most of the strain vanishes after fracture, indicating small plastic deformation. Poisson ratios close to one were observed in the RT plane, in agreement with macroscopic literature. Our work allowed for the first time to link this behavior to microstructural deformations at subcellular scale. In particular, we quantified an increase in the perimeter of the tracheid cell lumens, while the cell lumen surface was preserved. Accordingly, we observed that the cell wall thickness was subtly stretched (around 0.8% or 28 nm) to accommodate the lumen perimeter increase with the same cell wall substance.

Wood rays have been previously hypothesized as reinforcing elements in in-silico models and in isolated single-cell experimental studies. In this work, we were for the first time able to observe the mechanical deformation of wood rays embedded in solid wood. Strains were calculated independently for wood ray cells, and showed consistent distributions with tracheids, supporting the picture of a composite deforming as a single unit. On the other hand, we found several indications of the reinforcing role of wood rays in wood composite structure. These included a reduction of wood ray density at the fracture line, slightly lower local strains for wood rays than tracheids in radial direction, and a significant increase of local strain in regions where less wood rays were present. Both longitudinal and tangential Poisson compressive strains affected wood rays, reducing their lumen surface and the inner distances between lumens belonging to the same ray cluster. The embedding of wood rays and tracheids in solid wood microstructure introduces a geometric imbalance, with both cell types weaving around each other. This imbalance translates into shear strains $\varepsilon_{RT}$, which correlate with shifts in tracheid lumen orientation and wood ray cell inclination. In turn, wood ray inclination increases for larger adjacent tracheid lumens, and wood tracheid inclination increases with the presence of wood rays. Occurrence of fracture and larger strains correlated with smallest ray lumen sections. This supports the hypothesis of previous works that smaller wood ray sizes are associated with weaker mechanical properties.

Looking into softwood compressed longitudinally until densification, microstructural analysis provides sensitive features predicting composite failure. For instance, tracheid lumen area and shifts in cell inclination were early stage indicators of cell densification. Wood ray deformation is more distributed along the longitudinal direction than tracheids, showing that rays are less subject to densification. Overall, our work supports the accepted cellular wood model, in which rays act as stiff pins which reinforce the radial properties of the wood composite. On the other hand, we show that wood rays show measurable, albeit small, mechanical deformation within a loaded wood composite.

## Materials and methods

### Experiments

The SRμCT experiments were carried out at the TOMCAT (TOmographic Microscopy and Coherent rAdiology experimenTs) beamline of the SLS (Swiss Light Source), a third generation synchrotron light source located at the Paul Scherrer Institute in Villigen, Switzerland^26^ (Fig. 1a,b). Miniaturized wood specimens (Fig. 1d) were manufactured from defect-free Norway spruce (Picea abies Karst.) and loaded in tension for the radial direction (R-specimen) and in compression for the grain direction (L-specimen), while acquiring in situ SRμCT tomograms at successive deformation states^35,36^. In order to ensure failure within the observation window, both specimens were tapered at the center. The R-specimen (shown in Fig. 1d) was manufactured from a 30 × 5.7 × 2.3 mm^3^ plank and two-sided tapered to a minimum cross-section of 4.15 mm^2^, while the L-specimen consisted of an 8 mm long, 4 mm diameter cylinder, which was shaped into a hyperboloid of minimum 1.54 mm^2^ section. The density of the samples was 400 kg m^−3^ ± 60 kg m^−3^ and the moisture content 8% ± 0.3%. The moisture content (%) was determined by subtracting the dry oven mass of the specimens from the mass during measurements, and by dividing the difference by the oven-dry mass^76^. Twin specimens were manufactured for each of the synchrotron tested probes. The twin specimens were stored intact in the synchrotron station, and oven dried after the measurements. All specimens were pre-acclimatized to a temperature of 23 °C and a relative air humidity of 40% to achieve a nominal moisture content of 8%. The synchrotron measurement station was also acclimatized during the measurements to the same temperature and relative humidity conditions. Acoustic emission sensors were available within the synchrotron station, following the setup of Baensch et al.^36^. These sensors did not detect any signals, which revealed micro-fractures associated to drying processes during the measurements. The density was calculated with the gravimetric method as the ratio of mass to volume at acclimatized conditions, with the volume (width × length × thickness) measured for the plank before tapering.

A 1 kN loading device was custom designed for the tests^35^ (Fig. 1c). Tensile or compressive load are exerted by respectively pulling the sample downwards or upwards, while a fixed three-pieced plug system at the top of the sample functions as counterpart to close the force circuit. The center tube is made of a thermosetting polyamide-imide, which is characterized by low radiation absorption. The tube connects the loading device with the three-pieced plug system. For each loading step, the lower transverse drives at 0.01–0.05 mm/s a defined deformation into the sample, a 10 min stop time is imposed to minimize movement artifacts due to relaxation, and then a SRμCT tomogram is acquired. Each dataset consisted of 1501 scintigrams (2048 × 2048 px^2^) acquired at 0.12° steps in a 180° range. The R-specimen was measured at 20 keV beam energy using phase-contrast imaging (6 min/tomogram), and the L-specimen at 10 keV with absorption imaging (13 min/tomogram). The synchrotron imaging settings were adjusted empirically to maximize the sharpness of the tomograms, allowing visual discrimination of wood cell details, for instance, cell wall and wood pits. The settings for R- and L-specimens were adjusted independently by experienced synchrotron operators. The differences between configuration set choice for R-specimen and L-specimen are further related to the different data acquisition times and the different microstructure propagation path of the synchrotron beam in R- and L-configurations. In both cases a similar digital voxel size (1.62 μm and 1.8 μm, respectively) and a field of view (40 mm) comprising the full specimen section were achieved. The phase-contrast configuration is designed to enhance the resolution of materials with weak absorbance. It achieved slightly better effective lateral resolution (2.5 μm) than the absorption configuration (4 μm). The effective resolution was in both cases determined through modulation transfer function^24,77^.

### Individual cell tracking (ICT)

#### Automatic segmentation of tracheids and wood rays

The starting point is a SRμCT tomogram acquired in 8-bit grayscale (Fig. 1e,f). First, the tomoslice cross-sections were rotated so that the three axes (X, Y, Z) were coarsely aligned with the principal material axes (R, T, L). For both R- and L-specimens the load direction was respectively well-aligned with the R and L material axes. The datasets show two clearly differentiated phases^74^: cell wall substance (density ~ 1500 kg m^−3^) and lumen voids (air, density ~ 1.2 kg m^−3^), which are binarized using Otsu’s method^78^. For illustration of density profiles (for instance, Fig. 4b), the cell wall voxel density was normalized to 1300 ± 200 kg m^−3^, so that the average tomogram density in baseline (undeformed) state corresponded to the specimen’s gravimetric values. The effective cell wall density is lower than the nominal value (1500 kg m^−3^)^74^ due to the finite synchrotron lateral resolution. The segmentation of individual cells from the binary image $A_{x,y,z}$ was performed based on an efficient combination of three-dimensional morphological operations (opening, closing, opening by reconstruction, labelling of connected elements)^79^. The reference elements were the lumen voids, not the cell material, since they allow an easier segmentation into separate unconnected geometries. Cell lumens provide approximately closed tubular geometries, which are bounded by high-contrast grayscale gradients at the transitions between air and cell wall substance. Therefore, the datasets can be binarized and the cells can be well-separated with morphological image processing. The goal is to assign a cell category and a cell index to each voxel in the image, so that (1) a maximum number of tracheids are detected with minimum geometry distortion for an accurate deformation analysis, and (2) wood rays are correctly discriminated to analyze their influence in the deformation build up. To be able to measure the cell wall thickness for each wood cell, an additional step is necessary to assign cell wall substance to each corresponding wood cell lumen. The cell wall boundary—that is, the middle lamella^74^—does not provide enough contrast in synchrotron images to separate adjacent cells. Instead, a skeletonization step is carried out to assign cell wall material pixels to the geometrically closest cell lumen void. Details are described in Appendix A of the Supplementary Materials.

The segmentation operates fully automatically over large volume subsets (1.7 × 10^9^ px^3^ = 8 mm^3^ for R samples). The data evaluation was implemented in MATLAB (The Mathworks Inc., Natick MA, USA). An example of the performance of the segmentation of a binarized SRµCT dataset is shown in Fig. 2a. High resolution renders of wood tracheid lumens are shown in Fig. 2b. Segmented wood rays are shown in Fig. 2c, including clustering of wood ray lumens belonging to the same wood ray.

#### Tracheid deformation analysis

After segmentation, the goal of ICT is to estimate the displacement vector ${\mathbf{u}} = \left( {u_{x} ,u_{y} ,u_{z} } \right)^{T}$ at each voxel of the analyzed volume. For this, two datasets (reference #1, test #2) at two different deformation states are coarsely aligned in region of interest before applying the following steps:

*Cell registration* For each tracheid element in #1, find cell indexes of overlapping cells in #2 and their number of overlapping voxels. The overlap of #2 in #1 should be at least 50% of the volume of #1. The same condition is applied to the overlap of #1 in #2.

*Tracheid cell analysis* Wood tracheids are parametrized with a one-dimensional (1D) representation in function of Z (L) coordinate (Fig. 2b). For each tracheid and Z coordinate, 2D lumen sections (in XY = RT plane) with single connectivity and no inner voids are extracted from the segmentation. From each lumen section and Z coordinate we calculate a list of geometrical parameters ($c_{X}$: centroid X, $c_{Y}$: centroid Y, ${\Sigma }$: lumen cross-section area), including as well an elliptical fit of the 2D lumen ($e_{a}$: major axis, $e_{b}$: minor axis, $e_{{\Psi }}$: XY orientation). The average cell wall thickness $t$ is calculated as $t = 2\left( {{\Sigma }_{{{\text{sk}}}} - {\Sigma }} \right)/\left( {{\text{P}}_{{{\text{sk}}}} - {\text{P}}} \right)$, where ${\Sigma }_{{{\text{sk}}}}$ is the area of the skeletonized cell cross-section in 3.1 (including cell wall material). ${\text{P}}$ and ${\text{P}}_{{{\text{sk}}}}$ are respectively the perimeters of the lumen void and skeletonized cell cross-section. Finally, the cell inclination angle $\theta \left( z \right)$ with respect to the fiber direction $\theta \left( z \right) = \tan^{ - 1} \sqrt {\left( {\partial_{z} c_{X} } \right)^{2} + \left( {\partial_{z} c_{Y} } \right)^{2} }$ is estimated from the Z-derivatives of the centroid coordinates, which are calculated with *smoothing splines* with resolution of 50 px = 81 µm. The calculated inclination angle is used to reslice the tracheid volume and obtain lumen cross-sections perpendicular to the inclination vector, for which the geometrical parameters are recalculated. The procedure is repeated iteratively 3 times until the geometric parameters converge.

*Texture correlation of Z-deformation fields *The Z-deformation along the tracheid longitudinal axes L is calculated based on a one-dimensional implementation of the adaptive texture correlation algorithm described in^80^. Given the test $T$ and reference $T_{0}$ subsets, each containing the parametrized representation of a wood cell, the optimum Z-deformation $u_{z}$ maximizes the correlation between the list $i_{p} = \left\{ {c_{X} ,c_{Y} ,{{\Sigma }},{\text{P}},e_{a} ,e_{b} ,e_{{\Psi }} ,t,\theta } \right\}$ of lumen geometrical parameters at each *z* position*,* which is calculated with a zero-normalized cross-correlation function (ZNCC):

$$ I_{corr} \left( {z,\hat{z}} \right) = \frac{{\sum \nolimits_{ip} \sum \nolimits_{w \in W} \left[ {T\left( {z + w + \hat{z},i_{p} } \right) - \overline{T}} \right]\left[ {T_{0} \left( {z + w,i_{p} } \right) - \overline{T}_{0} } \right]}}{{ \sum \nolimits_{ip} \sum \nolimits_{w \in W} \left[ {T\left( {z + w + \hat{z},i_{p} } \right) - \overline{T}} \right]^{2} \sum \nolimits_{ip} \sum \nolimits_{w \in W} \left[ {T\left( {z + w,i_{p} } \right) - \overline{T}_{0} } \right]^{2} }} $$

$$ u_{z} \left( z \right) = \mathop {{\text{argmax}}}\limits_{{\hat{z}}} I_{corr} \left( {z,\hat{z}} \right) $$

The correlation subset is W = 41 px = 66 μm. $u_{z}$ estimates are accepted as reliable if $I_{corr}$ > 0.5.

*Computation of macroscopic deformation fields and cell geometry deformation *The calculated deformation fields $u_{z} \left( z \right)$ are used to align in Z the test parametrized cell with the reference cell $\hat{T}\left( {z,i_{p} } \right) = T\left( {z + u_{z} \left( z \right),i_{p} } \right)$ with bicubic interpolation. From the aligned datasets, the deformation fields in X and Y are calculated by subtracting the centroids $u_{x} \left( z \right) = \hat{T}\left( {z,c_{X} } \right) - T_{0} \left( {z,c_{X} } \right)$ and $u_{Y} \left( z \right) = \hat{T}\left( {z,c_{Y} } \right) - T_{0} \left( {z,c_{Y} } \right)$. The swelling of cell lumen area is estimated as $\varepsilon_{{\Sigma }} \left( z \right) = \left( {\hat{T}\left( {z,{\Sigma }} \right) - T_{0} \left( {z,{\Sigma }} \right)} \right)/T_{0} \left( {z,{\Sigma }} \right)$, the swelling of cell wall thickness as $\varepsilon_{{\text{t}}} \left( z \right) = \left( {\hat{T}\left( {z,{\text{t}}} \right) - T_{0} \left( {z,{\text{t}}} \right)} \right)/T_{0} \left( {z,{\text{t}}} \right)$, and the cell inclination change as ${\Delta }\theta \left( z \right) = \hat{T}\left( {z,\theta } \right) - T_{0} \left( {z,\theta } \right)$. Similar deformation parameters are defined for the fitted ellipse ($e_{a} ,e_{b} ,e_{{\Psi }}$). Selected deformation parameters are illustrated in Fig. 7 for a single tracheid loaded in compression.

*Macroscopic strain computation *The calculated cell deformation parameters provide readings of the deformation ${\mathbf{u}} = \left( {u_{x} ,u_{y} ,u_{z} } \right)^{T}$ at discrete coordinates $\left( {c_{x} ,c_{y} ,z} \right)^{T}$, corresponding to the centroids of the cell lumens along Z. Differentiable strain fields $\varepsilon_{ij} = 0.5\left( {\partial_{j} u_{i} + \partial_{i} u_{j} } \right)$ are estimated in two spline extrapolation steps. The Z smoothing is implemented with *cubic smoothing splines* (csaps function in MATLAB) and Y smoothing with *thin plate smoothing splines* (tpaps function in MATLAB)^81^. The latter allows for arbitrary data sites within a plane, corresponding to the centroid positions. The spline smoothness is given by a resolution parameter *h*, which is typically set to 50 px = 80 μm.

#### Wood ray deformation analysis

For wood rays, lumen cross-sections are 1D-parametrized as in 3.2. in function of the radial coordinate Z = R. Since the lumen diameter is smaller for wood rays (7 µm) than tracheids (35 µm), coarser Z correlation subsets and strain resolution (100 px) are used to reduce estimation noise. Additionally, affine strain $\varepsilon_{{{\text{ray}}}}$ is extracted from the LT displacements of the lumen centroids clustered to the same wood ray, and associated to linear swelling/compression of the wood ray and non-affine deformation $u_{{{\text{ray}}}}$ to plastic deformation and fracture (Fig. 2c). To correlate deformation fields with the presence of wood rays, the Euclidean distance transform of the segmented wood rays.

## Supplementary information

Supplementary Information.

## Data Availability

The authors provide complete correlation tables between macroscopic deformation, cell deformation and cell geometric parameters in the Supplementary Materials. Synchrotron Computed Tomography Datasets and implemented code for cell segmentation and Individual Cell Tracking are available upon reasonable requests.
