# Supplementary material for: In-situ quantification of microscopic contributions of individual cells to macroscopic wood deformation with synchrotron computed tomography
Source: Sci Rep. 2020 Dec 10;10:21615. doi: 10.1038/s41598-020-78028-4 (PMC7730398; doi:10.1038/s41598-020-78028-4)
Supplement: Supplementary file 1 — Supplementary Information. [file 41598_2020_78028_MOESM1_ESM.docx]

**In-situ quantification of microscopic contributions of individual cells to
macroscopic wood deformation with synchrotron computed tomography**

*Sanabria, Sergio J. (Sergio.Sanabria@usz.ch, T +41 44 255 43 69, F +41 44 255 44 43)^a^; Baensch, Franziska^b^; Zauner, Michaela^c^; Niemz, Peter^c^

^a^Institute of Diagnostic and Interventional Radiology, University Hospital Zurich, Raemistrasse 100, CH-8091 Zurich

^b^Federal Institute for Materials Research and Testing (German: BAM), Unter den Eichen 87, DE-112205 Berlin

^c^Institute for Building Materials, ETH Zurich, Stefano-Franscini-Platz 6, CH-8093 Zurich

**Supplementary materials**

**Appendix A. Segmentation of tracheids and wood rays**

The starting point of the segmentation is a binary 3D dataset _
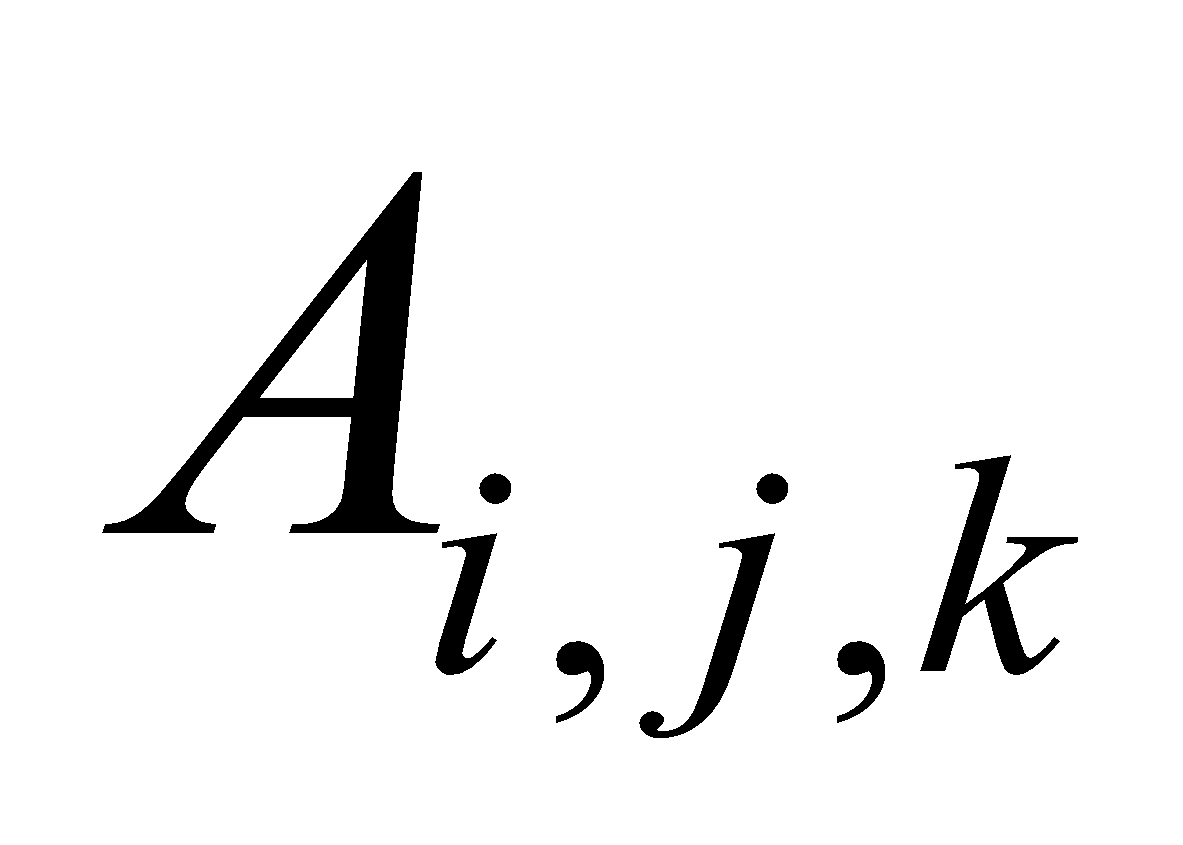
_, where “1”s correspond to cell wall substance and “0”s to cell lumen voids.

***Large amorphous regions*** (resin canals, glue lines) with relatively homogeneous X-ray absorption and low contrast with respect the cell substance are delineated and extracted from the images as a pre-step to tracheid/ray segmentation by binarizing the gradient image and performing a morphological opening operation with a spheroid kernel (radius 6 px = 10 µm), which removes the connections with the surrounding cell walls.

***Tracheids*** are detected by opening $A^{s}\circ s_{\text{Line}_{L}}$ the complement image $A^{s}$ with a line element in L direction (length 12 px = 19 µm), which breaks up pit connections between adjacent tracheids. The resulting elements are individually labelled and a minimum size (40 px = 65 µm in L and 5 px = 8 µm in R and T) is imposed to their bounding box, which allows filtering out wood rays and small fragments. *Edge elements,* that is, cells in contact with dataset boundaries are excluded from the evaluation. Next, *connected tracheid elements*, that is, two or more adjacent tracheids wrongly segmented as one, are identified by labelling and counting the number of unconnected two-dimensional voids in cross-sections along L. A well segmented tracheid ideally shows a single void cross-section. Connected tracheid elements appear when the resolution falls below the cell diameter, for instance in the case of latewood cells with small lumen sizes. Tracheids for which unconnected voids are found in at least 25% of the RT sections are classified as connected. In the remaining cells, for each L cross-section showing multiple unconnected voids, the smallest voids (up to 25% of the total void size) are filtered out. A larger line opening in L (24 px = 39 µm), followed by a circular disk opening $A^{s}\circ s_{\text{Disk}_{RT}}$ in the RT plane (radius 3 px = 5 µm) is then used to separate the connected tracheids. The *milling noise* (lumen connections filtered out by the opening operations) is labelled as a separate voxel category. Next, the connectivity analysis is repeated.

***Wood rays*** are detected from the remaining cell lumen data after filtering out already segmented amorphous regions, tracheid elements, edge elements, connected tracheid elements and milling noise. The wood rays are composed of multiple elongated pore structures in R, which fit onto well-aligned lines in R along the full ray length. Multiple parallel such lines appear adjacently in L (uniseriate wood rays) and in occasions T (multi-seriate wood rays). Pit connections between uniseriate/multiseriate wood ray lumens are eliminated by first opening the image $A^{s}\circ s_{\text{Line}_{R}}$ with a line element in R (length 3 px = 5 µm). Ray cell lumens are then separated from other cell structures by filtering out from the dataset morphological openings $A\wedge\left( A^{s}\circ s_{\text{Line}_{\left\{ L,T \right\}}} \right)^{s}$ with line elements in both L and T direction, which are longer than (30 px = 49 µm). Next the binary images are filtered in R with a square window $A^{s}*s_{\text{Line}_{R}}$ (length 50 px = 81 µm), which is used to detect voxels $\left( A^{s}*s_{\mathrm{Line}_{R}} \right)>0.5\cdot sum\left\{ s_{\mathrm{Line}_{R}} \right\}$, in which the $s_{\text{Line}_{R}}$ neighborhood contains at least 50% of cell lumen voxels. These voxels are used as seed points for the morphological reconstruction of ray lumen voids from $A^{s}$. The detected ray lumen voids are then clustered as belonging to the same ray. The clustering mask is calculated by applying to the detected ray pores morphological closing in both R, L and T directions $A^{s}\circ s_{\text{Line}_{R}}\circ s_{\text{Line}_{L}}\circ s_{\text{Line}_{T}}$ (10 px = 16 µm in L and T, and 25 px = 41 µm in R). A minimum cluster size in R (100 px = 162 µm) imposes a minimum ray cell length.

***Segmentation of cell wall:*** A skeletonization by influence zones (SKIZ)^1^ step is finally carried out, which distributes and assigns the cell wall material to the previously segmented cell lumens – air voids. This allows estimating and segmenting the cell wall boundaries associated to a particular lumen. Prior to skeletonization, the *milling* noise is also then associated to the spatially closest elements, so that wood pits can be used as landmarks in the L direction for the final segmented cells. The zone of influence of each cell lumen is defined as the set of points of the cell wall that are closer to this cell lumen than to any other component. SKIZ is calculated as the boundary of all zones of influence, which effectively estimates the position of the middle lamella of the cell wall for each lumen. SKIZ was implemented in MATLAB using an Euclidean distance transform (bwdist command) followed by a watershed segmentation step (watershed command)^1^. For each voxel of the cell wall, the Euclidean distance to the closest air lumen is calculated^2^. The watershed transform^3^ then calculates the cell wall boundaries as “catchment basins” or “watershed ridge lines” in the distance transform. An example of segmentation and skeletonization for the R-specimen is shown in Figure S1.


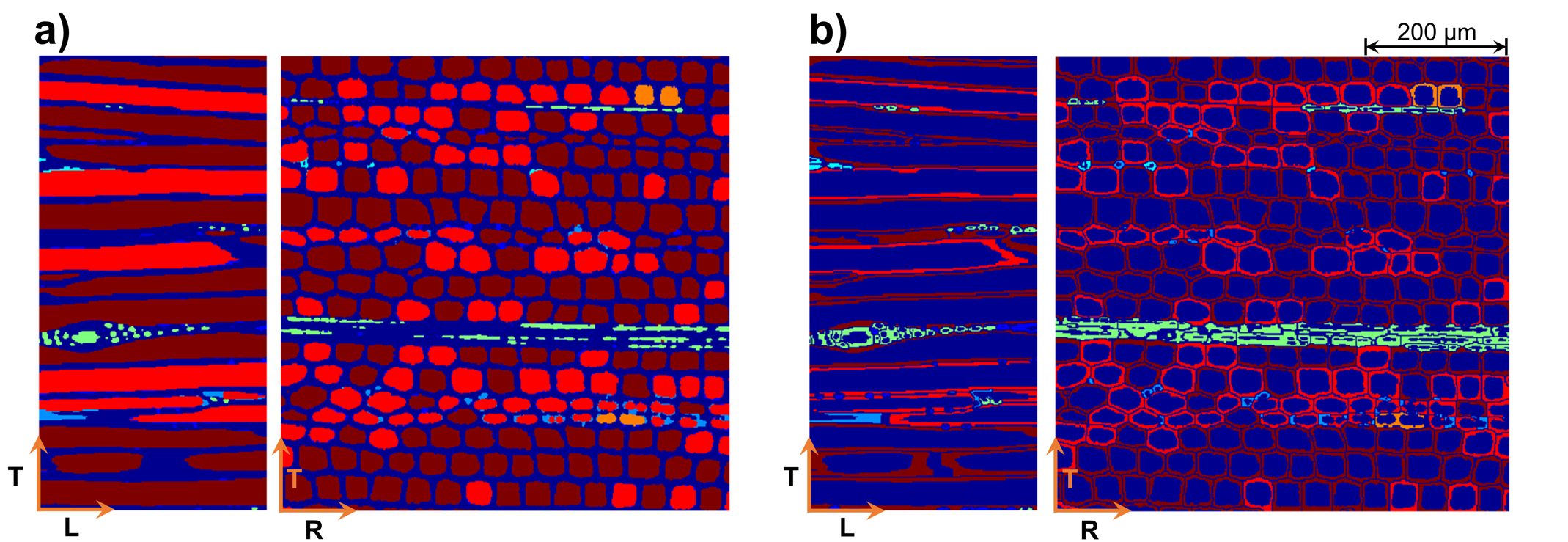


Figure S1: Automatic cell segmentation. a) Segmentation of tracheids (red tones) and wood rays (green tones). Unassigned cell fragments are plotted in cyan tones. The morphological segmentation operates on the cell lumen voids (colored), which show closed geometries. The cell wall substance (dark blue) does not show synchrotron contrast at the middle lamella for binarization. b) Skeletonization by influence zones (SKIZ) segmentation of cell wall. The cell wall voxels are distributed among the adjacent cell lumens according to spatial proximity.

**Appendix B. Correlation tables between macroscopic deformation, cell deformation and cell geometric parameters**

The outputs of the Individual Cell Tracking algorithm are three-dimensional distributions of strain and geometrical (e.g. lumen area, cell inclination) parameters. Correlations between parameters are obtained voxel-wise in terms of Pearson coefficient $r$, with p-value less than 0.05 considered significant. The strongest correlations are visually highlighted with color shading (darker: stronger correlation), with green for positive correlations ($r>0)$and red for negative correlations $(r<0)$.

Table S1 – Correlation coefficient *r* between wood tracheid deformation parameters. All $r>0.001$ satisfy the significance criterion p<$0.05$. $\varepsilon_{ij}$ are macroscopic strains for wood anatomical directions (R: radial, T: tangential, L: grain). The remaining parameters are cellular geometry values: lumen-cross sectional area $\Sigma$ and swelling $\varepsilon_{\Sigma}$; fitted lumen ellipse parametrized by major axis $e_{a}$, minor axis $e_{b}$ and orientation $e_{\Psi}$ , with corresponding swelling $\varepsilon_{ea}$, $\varepsilon_{eb}$ and orientation shift ${\Delta e}_{\Psi}$; cell inclination $\theta$ and inclination shift $\Delta\theta$; cell wall thickness $t$ and swelling $e_{t}$.

|  | $\varepsilon_{RR}$ | $\varepsilon_{TT}$ | $\varepsilon_{LL}$ | $\varepsilon_{TL}$ | $\varepsilon_{LR}$ | $\varepsilon_{TR}$ | $\Sigma$ | $\varepsilon_{\Sigma}$ | $e_{a}$ | $\varepsilon_{ea}$ | $e_{b}$ | $\varepsilon_{eb}$ | $e_{\Psi}$ | Δ$e_{\Psi}$ | $\theta$ | $\Delta\theta$ | $t$ | $\varepsilon_{t}$ |
| --- | --- | --- | --- | --- | --- | --- | --- | --- | --- | --- | --- | --- | --- | --- | --- | --- | --- | --- |
| $\varepsilon_{RR}$ | 1.000 | -0.639 | 0.003 | -0.028 | 0.075 | 0.225 | 0.166 | -0.124 | 0.154 | 0.284 | 0.177 | -0.373 | -0.124 | -0.008 | 0.179 | -0.046 | -0.229 | -0.089 |
| $\varepsilon_{TT}$ |  | 1.000 | -0.008 | -0.037 | 0.005 | -0.140 | -0.262 | 0.251 | -0.340 | -0.306 | -0.183 | 0.533 | 0.064 | -0.060 | -0.222 | 0.005 | 0.457 | 0.083 |
| $\varepsilon_{LL}$ |  |  | 1.000 | -0.035 | 0.033 | 0.020 | 0.031 | -0.020 | 0.035 | 0.013 | 0.021 | -0.037 | -0.033 | -0.034 | 0.024 | -0.031 | -0.021 | -0.027 |
| $\varepsilon_{TL}$ |  |  |  | 1.000 | -0.006 | -0.012 | -0.008 | 0.012 | -0.005 | -0.011 | -0.003 | 0.023 | -0.001 | 0.009 | -0.031 | 0.017 | 0.044 | 0.001 |
| $\varepsilon_{LR}$ |  |  |  |  | 1.000 | 0.003 | 0.005 | 0.024 | 0.002 | 0.017 | 0.008 | 0.013 | 0.009 | -0.011 | -0.010 | 0.043 | 0.007 | -0.020 |
| $\varepsilon_{TR}$ |  |  |  |  |  | 1.000 | 0.109 | 0.055 | 0.100 | 0.198 | 0.114 | -0.110 | -0.041 | -0.312 | 0.018 | -0.067 | -0.100 | -0.094 |
| $\Sigma$ |  |  |  |  |  |  | 1.000 | -0.170 | 0.888 | 0.116 | 0.918 | -0.278 | -0.044 | 0.030 | 0.152 | 0.052 | -0.332 | -0.007 |
| $\varepsilon_{\Sigma}$ |  |  |  |  |  |  |  | 1.000 | -0.239 | 0.495 | -0.084 | 0.711 | 0.033 | -0.099 | -0.129 | 0.037 | 0.264 | -0.344 |
| $e_{a}$ |  |  |  |  |  |  |  |  | 1.000 | 0.167 | 0.662 | -0.405 | -0.030 | 0.045 | 0.173 | 0.050 | -0.481 | 0.000 |
| $\varepsilon_{ea}$ |  |  |  |  |  |  |  |  |  | 1.000 | 0.070 | -0.237 | -0.071 | -0.023 | 0.040 | 0.014 | -0.131 | -0.352 |
| $e_{b}$ |  |  |  |  |  |  |  |  |  |  | 1.000 | -0.143 | -0.044 | 0.006 | 0.093 | 0.050 | -0.180 | -0.017 |
| $\varepsilon_{eb}$ |  |  |  |  |  |  |  |  |  |  |  | 1.000 | 0.093 | -0.089 | -0.185 | 0.031 | 0.421 | -0.116 |
| $e_{\Psi}$ |  |  |  |  |  |  |  |  |  |  |  |  | 1.000 | -0.247 | -0.063 | 0.007 | 0.024 | 0.047 |
| Δ$e_{\Psi}$ |  |  |  |  |  |  |  |  |  |  |  |  |  | 1.000 | 0.039 | 0.018 | -0.071 | 0.026 |
| $\theta$ |  |  |  |  |  |  |  |  |  |  |  |  |  |  | 1.000 | -0.070 | -0.459 | 0.024 |
| $\Delta\theta$ |  |  |  |  |  |  |  |  |  |  |  |  |  |  |  | 1.000 | -0.057 | -0.018 |
| $t$ |  |  |  |  |  |  |  |  |  |  |  |  |  |  |  |  | 1.000 | -0.143 |
| $\varepsilon_{t}$ |  |  |  |  |  |  |  |  |  |  |  |  |  |  |  |  |  | 1.000 |

Table S2 – Correlation between wood ray deformation parameters. All $r>0.001$ satisfy the significance criterion p<$0.05$. $\varepsilon_{ij}$ are macroscopic strains for wood anatomical directions (R: radial, T: tangential, L: grain). Apart from the parameters defined in Table B.1, ray cluster strain $\varepsilon_{\mathrm{ray}}$ and distance to closest wood ray cell $d_{\mathrm{ray}}$ are introduced.

|  | $\varepsilon_{RR}$ | $\varepsilon_{TT}$ | $\varepsilon_{LL}$ | $\varepsilon_{TL}$ | $\varepsilon_{LR}$ | $\varepsilon_{TR}$ | $\Sigma$ | $\varepsilon_{\Sigma}$ | $\varepsilon_{ray}$ | $e_{a}$ | $\varepsilon_{ea}$ | $e_{b}$ | $\varepsilon_{eb}$ | $e_{\Psi}$ | Δ$e_{\Psi}$ | $\theta$ | $\Delta\theta$ | $d_{ray}$ |
| --- | --- | --- | --- | --- | --- | --- | --- | --- | --- | --- | --- | --- | --- | --- | --- | --- | --- | --- |
| $\varepsilon_{RR}$ | 1.000 | 0.008 | 0.039 | 0.289 | -0.047 | 0.143 | 0.167 | 0.092 | -0.087 | 0.181 | 0.097 | 0.148 | 0.069 | 0.073 | -0.116 | 0.209 | -0.030 | -0.037 |
| $\varepsilon_{TT}$ |  | 1.000 | 0.372 | 0.238 | -0.058 | -0.212 | 0.562 | 0.112 | 0.384 | 0.555 | 0.034 | 0.511 | 0.194 | -0.142 | -0.156 | 0.073 | 0.250 | -0.081 |
| $\varepsilon_{LL}$ |  |  | 1.000 | 0.225 | -0.090 | -0.105 | 0.255 | 0.237 | 0.259 | 0.259 | 0.147 | 0.192 | 0.290 | -0.043 | -0.071 | 0.101 | 0.125 | -0.019 |
| $\varepsilon_{TL}$ |  |  |  | 1.000 | -0.071 | 0.191 | 0.217 | 0.071 | 0.185 | 0.251 | 0.002 | 0.157 | 0.126 | 0.070 | 0.027 | 0.505 | -0.162 | -0.022 |
| $\varepsilon_{LR}$ |  |  |  |  | 1.000 | 0.068 | -0.059 | -0.052 | 0.046 | -0.072 | -0.038 | -0.056 | -0.040 | 0.003 | 0.064 | -0.001 | -0.024 | 0.035 |
| $\varepsilon_{TR}$ |  |  |  |  |  | 1.000 | -0.175 | 0.043 | -0.017 | -0.130 | 0.115 | -0.211 | -0.059 | 0.274 | 0.072 | -0.032 | -0.382 | 0.004 |
| $\Sigma$ |  |  |  |  |  |  | 1.000 | 0.112 | 0.261 | 0.970 | 0.017 | 0.969 | 0.218 | -0.241 | -0.137 | 0.382 | 0.319 | -0.147 |
| $\varepsilon_{\Sigma}$ |  |  |  |  |  |  |  | 1.000 | 0.186 | 0.069 | 0.866 | 0.099 | 0.893 | 0.101 | -0.430 | 0.038 | 0.017 | -0.048 |
| $\varepsilon_{ray}$ |  |  |  |  |  |  |  |  | 1.000 | 0.260 | 0.106 | 0.231 | 0.234 | -0.041 | -0.213 | 0.065 | 0.006 | -0.062 |
| $e_{a}$ |  |  |  |  |  |  |  |  |  | 1.000 | -0.031 | 0.897 | 0.190 | -0.181 | -0.130 | 0.350 | 0.278 | -0.146 |
| $\varepsilon_{ea}$ |  |  |  |  |  |  |  |  |  |  | 1.000 | 0.012 | 0.573 | 0.135 | -0.348 | -0.017 | -0.053 | -0.036 |
| $e_{b}$ |  |  |  |  |  |  |  |  |  |  |  | 1.000 | 0.195 | -0.278 | -0.119 | 0.394 | 0.338 | -0.140 |
| $\varepsilon_{eb}$ |  |  |  |  |  |  |  |  |  |  |  |  | 1.000 | 0.028 | -0.393 | 0.100 | 0.098 | -0.051 |
| $e_{\Psi}$ |  |  |  |  |  |  |  |  |  |  |  |  |  | 1.000 | -0.218 | -0.285 | -0.074 | -0.001 |
| Δ$e_{\Psi}$ |  |  |  |  |  |  |  |  |  |  |  |  |  |  | 1.000 | 0.027 | -0.109 | 0.060 |
| $\theta$ |  |  |  |  |  |  |  |  |  |  |  |  |  |  |  | 1.000 | -0.144 | -0.037 |
| $\Delta\theta$ |  |  |  |  |  |  |  |  |  |  |  |  |  |  |  |  | 1.000 | -0.060 |
| $d_{ray}$ |  |  |  |  |  |  |  |  |  |  |  |  |  |  |  |  |  | 1.000 |

Table S3 – Correlation between wood tracheid (rows) and wood ray (columns) deformation parameters. All $r>0.001$ satisfy the significance criterion p<$0.05$.

|  | $\varepsilon_{RR}$ | $\varepsilon_{TT}$ | $\varepsilon_{LL}$ | $\varepsilon_{TL}$ | $\varepsilon_{LR}$ | $\varepsilon_{TR}$ | $\Sigma$ | $\varepsilon_{\Sigma}$ | $\varepsilon_{ray}$ | $e_{a}$ | $\varepsilon_{ea}$ | $e_{b}$ | $\varepsilon_{eb}$ | $e_{\Psi}$ | Δ$e_{\Psi}$ | $\theta$ | $\Delta\theta$ | $d_{ray}$ |
| --- | --- | --- | --- | --- | --- | --- | --- | --- | --- | --- | --- | --- | --- | --- | --- | --- | --- | --- |
| $\varepsilon_{RR}$ | 0.133 | -0.698 | -0.246 | -0.052 | -0.010 | 0.244 | -0.355 | -0.103 | -0.261 | -0.336 | -0.059 | -0.324 | -0.159 | 0.125 | 0.113 | 0.007 | -0.190 | 0.052 |
| $\varepsilon_{TT}$ | -0.025 | 0.835 | 0.306 | 0.184 | -0.035 | -0.228 | 0.423 | 0.076 | 0.337 | 0.426 | -0.001 | 0.368 | 0.150 | -0.151 | -0.157 | 0.057 | 0.179 | -0.108 |
| $\varepsilon_{LL}$ | -0.007 | -0.004 | -0.033 | 0.016 | 0.001 | 0.011 | 0.005 | 0.006 | -0.042 | 0.004 | 0.015 | 0.007 | -0.009 | 0.006 | 0.019 | 0.030 | -0.024 | 0.003 |
| $\varepsilon_{TL}$ | 0.002 | 0.027 | -0.009 | 0.007 | -0.003 | -0.033 | 0.024 | -0.033 | -0.005 | 0.021 | -0.035 | 0.025 | -0.018 | -0.025 | 0.002 | -0.003 | 0.026 | 0.021 |
| $\varepsilon_{LR}$ | 0.004 | 0.011 | -0.019 | 0.006 | 0.012 | 0.007 | -0.010 | -0.004 | -0.024 | -0.012 | 0.007 | -0.007 | -0.019 | -0.001 | 0.007 | -0.003 | -0.017 | -0.001 |
| $\varepsilon_{TR}$ | 0.079 | -0.196 | -0.141 | 0.192 | 0.056 | 0.703 | -0.231 | 0.028 | 0.014 | -0.181 | 0.095 | -0.264 | -0.068 | 0.251 | 0.075 | -0.030 | -0.512 | 0.016 |
| $\Sigma$ | -0.104 | -0.390 | -0.204 | -0.112 | 0.116 | -0.039 | -0.212 | -0.052 | -0.237 | -0.217 | 0.000 | -0.169 | -0.070 | -0.162 | 0.119 | 0.142 | -0.175 | 0.059 |
| $\varepsilon_{\Sigma}$ | 0.132 | 0.249 | 0.083 | 0.202 | -0.040 | 0.055 | 0.155 | 0.006 | 0.091 | 0.167 | -0.021 | 0.128 | 0.020 | 0.034 | -0.101 | 0.096 | 0.019 | -0.056 |
| $e_{a}$ | -0.141 | -0.441 | -0.217 | -0.103 | 0.151 | -0.044 | -0.221 | 0.015 | -0.228 | -0.250 | 0.059 | -0.163 | -0.006 | -0.085 | 0.105 | 0.144 | -0.136 | 0.053 |
| $\varepsilon_{ea}$ | 0.096 | -0.321 | -0.108 | 0.109 | 0.005 | 0.192 | -0.171 | -0.009 | -0.100 | -0.177 | 0.013 | -0.149 | -0.045 | 0.084 | 0.059 | 0.106 | -0.168 | 0.050 |
| $e_{b}$ | -0.086 | -0.317 | -0.183 | -0.099 | 0.074 | -0.016 | -0.254 | -0.133 | -0.213 | -0.227 | -0.073 | -0.230 | -0.150 | -0.166 | 0.114 | 0.086 | -0.208 | 0.063 |
| $\varepsilon_{eb}$ | 0.080 | 0.542 | 0.179 | 0.137 | -0.053 | -0.098 | 0.322 | 0.010 | 0.179 | 0.340 | -0.038 | 0.274 | 0.058 | -0.040 | -0.162 | 0.024 | 0.158 | -0.101 |
| $e_{\Psi}$ | -0.078 | 0.105 | -0.030 | -0.015 | 0.063 | -0.019 | -0.011 | -0.077 | 0.037 | -0.012 | -0.042 | -0.016 | -0.073 | 0.003 | 0.051 | -0.110 | 0.031 | 0.014 |
| Δ$e_{\Psi}$ | -0.018 | -0.050 | -0.006 | -0.105 | 0.010 | -0.237 | 0.017 | -0.027 | -0.056 | -0.007 | -0.034 | 0.043 | -0.002 | -0.111 | -0.003 | 0.036 | 0.157 | 0.016 |
| $\theta$ | -0.026 | -0.255 | -0.037 | -0.070 | 0.054 | 0.035 | -0.155 | 0.049 | -0.071 | -0.148 | 0.050 | -0.146 | 0.037 | 0.041 | 0.084 | 0.043 | -0.012 | -0.060 |
| $\Delta\theta$ | -0.024 | -0.023 | -0.009 | -0.164 | 0.007 | -0.091 | -0.013 | 0.043 | -0.025 | -0.027 | 0.050 | -0.007 | 0.021 | -0.031 | -0.088 | -0.097 | 0.038 | -0.031 |
| $t$ | 0.152 | 0.527 | 0.188 | 0.171 | -0.103 | -0.024 | 0.383 | -0.058 | 0.145 | 0.398 | -0.094 | 0.330 | -0.014 | -0.051 | -0.087 | 0.020 | 0.129 | 0.025 |
| $\varepsilon_{t}$ | -0.109 | 0.077 | 0.055 | -0.086 | 0.034 | -0.101 | 0.022 | -0.062 | 0.031 | 0.036 | -0.059 | 0.006 | -0.037 | -0.058 | 0.035 | -0.072 | 0.057 | -0.028 |

**Appendix C. Contrast-To-Noise calculation for visualization of densification lines in Figure 7.**

To further verify the presence of the densification lines F1 and F2 in Fig.7, we summarize the deformation field indications: $\varepsilon_{zz}$<0 and $\varepsilon_{\sum}$<0 at the compression plane into a single Contrast To Noise Ratio (CNR) metric (section 2.3). CNR shows maxima at potential densification lines^4^:

$$CNR(L)=\frac{\left| \min_{\left[ L-W/2,L+W/2 \right]} \varepsilon\Sigma\right|+\left| \min_{\left[ L-W/1,L+W/+ \right]} \varepsilon_{LL} \right|}{\mathrm{std}\left( \varepsilon\Sigma\right)+std\left( \varepsilon_{LL} \right)}$$

W is the peak search window, which is set to the strain resolution of 50 px (section 4.1.2). Std is the standard deviation of noise in $\varepsilon_{zz}$ and $\varepsilon_{\sum}$ profiles – measured at the L coordinate range 1600 to 1700 µm, where no densification is present. The results are shown in Fig. S2. The peak CNR of 11.5 is observed at the densification line F1, followed by a second peak CNR of 4.7 at the secondary densification line F2. In-between F1 and F2, a less dominant relative maxima region with CNR of 3.8 corresponds to a third intermediate plane connecting F1 and F2.


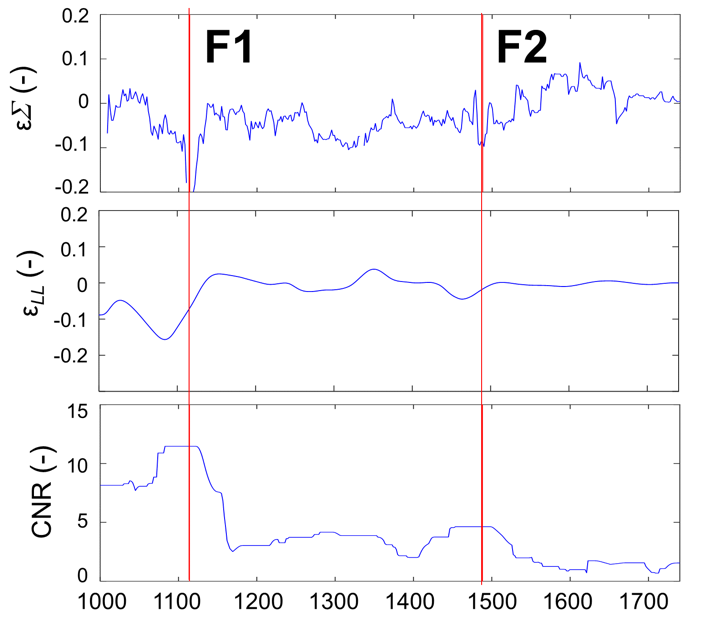
 Figure S2: Contrast-To-Noise calculation for deformation fields in Figure 7.

**Bibliography**

1. Preteux, E. Watershed and skeleton by influence zones: A distance-based approach. *J. Math. Imaging Vis.* **1**, 239–255 (1992).

2. Maurer, C., Rensheng, Q. & Raghavan, V. A linear time algorithm for computing exact Euclidean distance transforms of binary images in arbitrary dimensions. *IEEE Trans. Pattern Anal. Mach. Intell* **25**, 265–270 (2003).

3. Meyer, F. Topographic distance and watershed lines. *Signal Processing* **38**, 113–125 (1994).

4. Sanabria, S. J., Wyss, P., Neuenschwander, J., Niemz, P. & Sennhauser, U. Assessment of glued timber integrity by limited-angle microfocus X-ray computed tomography. *Eur. J. Wood Wood Prod.* **69**, 605–617 (2011).
